# Rapid acceleration of *KRAS-*mutant pancreatic carcinogenesis via remodeling of tumor immune microenvironment by PPARδ

**DOI:** 10.1038/s41467-022-30392-7

**Published:** 2022-05-13

**Authors:** Yi Liu, Yasunori Deguchi, Daoyan Wei, Fuyao Liu, Micheline J. Moussalli, Eriko Deguchi, Donghui Li, Huamin Wang, Lovie Ann Valentin, Jennifer K. Colby, Jing Wang, Xiaofeng Zheng, Haoqiang Ying, Mihai Gagea, Baoan Ji, Jiaqi Shi, James C. Yao, Xiangsheng Zuo, Imad Shureiqi

**Affiliations:** 1 Department of Gastrointestinal Medical Oncology, The University of Texas MD Anderson Cancer Center, Houston, TX 77030 USA; 2 Department of Gastroenterology, Hepatology, and Nutrition, The University of Texas MD Anderson Cancer Center, Houston, TX 77030 USA; 3 Department of Palliative, Rehabilitation, and Integrative Medicine, The University of Texas MD Anderson Cancer Center, Houston, TX 77030 USA; 4 Rogel Cancer Center and Department of Internal Medicine, Division of Hematology and Oncology, University of Michigan, Ann Arbor, MI 48109 USA; 5 Department of Pathology, The University of Texas MD Anderson Cancer Center, Houston, TX 77030 USA; 6 Department of Bioinformatics and Computational Biology, The University of Texas MD Anderson Cancer Center, Houston, TX 77030 USA; 7 Department of Molecular and Cellular Oncology, The University of Texas MD Anderson Cancer Center, Houston, TX 77030 USA; 8 Department of Veterinary Medicine and Surgery, The University of Texas MD Anderson Cancer Center, Houston, TX 77030 USA; 9 Department of Cancer Biology, Mayo Clinic, Jacksonville, FL 32224 USA; 10 Department of Pathology, University of Michigan, Ann Arbor, MI 48109 USA

**Keywords:** Cancer microenvironment, Pancreatic cancer, Cancer models, Cancer metabolism, Cancer genomics

## Abstract

Pancreatic intraepithelial neoplasia (PanIN) is a precursor of pancreatic ductal adenocarcinoma (PDAC), which commonly occurs in the general populations with aging. Although most PanIN lesions (PanINs) harbor oncogenic *KRAS* mutations that initiate pancreatic tumorigenesis; PanINs rarely progress to PDAC. Critical factors that promote this progression, especially targetable ones, remain poorly defined. We show that peroxisome proliferator-activated receptor-delta (PPARδ), a lipid nuclear receptor, is upregulated in PanINs in humans and mice. Furthermore, PPARδ ligand activation by a high-fat diet or GW501516 (a highly selective, synthetic PPARδ ligand) in mutant *KRAS*^*G12D*^ (*KRAS*^*mu*^) pancreatic epithelial cells strongly accelerates PanIN progression to PDAC. This PPARδ activation induces *KRAS*^*mu*^ pancreatic epithelial cells to secrete CCL2, which recruits immunosuppressive macrophages and myeloid-derived suppressor cells into pancreas via the CCL2/CCR2 axis to orchestrate an immunosuppressive tumor microenvironment and subsequently drive PanIN progression to PDAC. Our data identify PPARδ signaling as a potential molecular target to prevent PDAC development in subjects harboring PanINs.

## Abstract (web summary)

Pancreatic intraepithelial neoplasia (PanIN) can develop into pancreatic ductal adenocarcinoma (PDAC), however, the factors which determine how this occurs are unknown. Here, the authors illustrate the role of PPARδ in the upregulation of CCL2, resulting in an immunosuppressive microenvironment, and driving the progression of PanIN to PDAC.

## Introduction

Identification of critical factors that promote the progression of pancreatic intraepithelial neoplasia (PanIN) to pancreatic ductal adenocarcinoma (PDAC) could lead to the development of new interventions to reduce the rising incidence of PDAC^1^. PanINs are precursors for the vast majority of PDACs^2^. Approximately 54–78% of adults older than 40 years are estimated to harbor low-grade PanINs^3,4^; fortunately, the majority do not develop PDAC^5^. *KRAS* mutations are initiating events in pancreatic tumorigenesis and occur in more than 90% of PanINs^6^. However, the progression of PanIN to PDAC requires additional factors beyond *KRAS* mutations^7^ that need to be identified.

High-fat diets (HFDs) have been associated with increased risk for human PDAC^8–10^. In preclinical animal models, HFDs promotes both chemically induced and mutant *Kras*^*G12D*^ (*KRAS*^*mu*^)*-*initiated pancreatic tumorigenesis^11–18^. HFDs are enriched with fatty acids that act as activating natural ligands of peroxisome proliferator-activated receptor-delta (PPARδ, encoded by *PPARD*). PPARδ, as a transcriptional receptor, regulates the expression of a wide spectrum of key genes influencing important molecular events (e.g., lipid metabolism and tumorigenesis)^19^. PPARδ is upregulated in major human cancers, including pancreatic cancers^20–23^. PPARδ upregulation in human pancreatic cancers correlates with higher pathological stages and a higher risk of metastasis^22^. However, the effects of PPARδ on pancreatic tumorigenesis, especially in relation to HFDs, remain poorly defined. We found that PPARδ in Panc02 mouse PDAC cells strongly promoted pancreatic cancer metastasis^24^. However, others have proposed that PPARδ upregulation in human PDAC cells might be only a stress response indicator rather than being “causally related to the tumor phenotype”, based on in vitro study results showing that a selective PPARδ synthetic agonist, GW501516, inhibited human PDAC cell migration^25^. Thus, the mechanistic role of PPARδ in pancreatic carcinogenesis remains controversial, and further studies are needed to address this knowledge gap.

Selective, synthetic PPARδ agonists (e.g., GW501516) were initially developed and tested clinically to treat non-cancerous metabolic disorders (e.g., obesity)^26,27^. However, large pharmaceutical companies abandoned the development of PPARδ agonists because of concerns regarding their carcinogenic effects. Nonetheless, PPARδ agonists such as GW501516 are still illicitly being sold to athletes to enhance muscle endurance via websites claiming a lack of evidence for harmful effects. Given the availability of and uncertainty around these PPARδ agonists, there is a critical need to clarify the role of PPARδ in carcinogenesis.

We show in this work that PPARδ is upregulated in human and mouse PanINs and activation of PPARδ by its natural (e.g., fatty acids) or synthetic (e.g., GW501516) ligands in pancreatic epithelial cells carrying mutant *Kras*^*G12D*^ accelerates pancreatic carcinogenesis.

## Results

### PPARδ expression is upregulated in human and mouse PanIN lesions and regulated by *KRAS*^*mu*^ activity

**Fig. 1:**
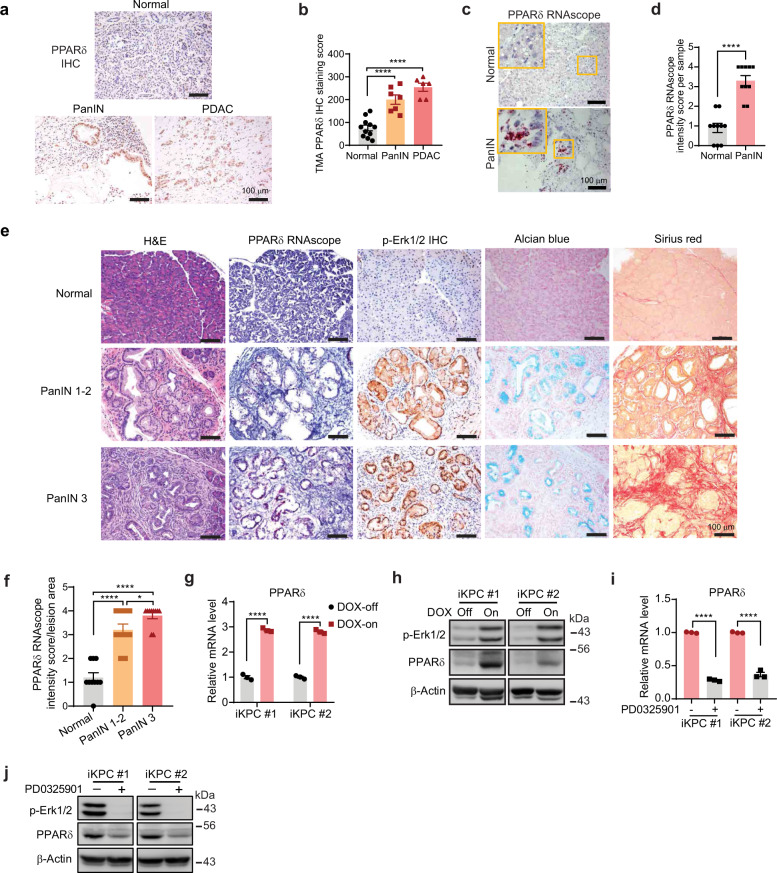
PPARδ expression is upregulated in human and mouse PanINs and regulated by *KRAS*^*mu*^ activity. **a**, **b** PPARδ protein expression in human normal, PanIN 1-2, and PDAC pancreatic tissues in a tissue microarray (TMA), measured by immunohistochemistry (IHC). **a** Representative images of PPARδ IHC staining of the indicated groups of human pancreatic tissue samples. **b** PPARδ IHC scores of normal (*n* = 11), PanIN 1-2 (*n* = 7), and PDAC (*n* = 6) pancreatic tissues obtained from TMA. **c**, **d** PPARδ mRNA expression levels in human normal and paired PanIN 1-2 (*n* = 10), measured by RNAscope in situ hybridization. **c** Representative images of PPARδ RNAscope in situ hybridization staining of the slides. Positive PPARδ mRNA staining is shown in red dots. **d** Quantitative results of PPARδ intensity scores. **e** Representative images of hematoxylin and eosin (H&E) staining, PPARδ RNAscope in situ hybridization, phospho-Erk1/2 (p-Erk1/2) IHC staining, Alcian blue staining, and Sirius red staining of normal, PanIN 1-2, and PanIN 3 pancreatic tissues in KC mice. **f** Quantitative data of PPARδ mRNA intensity scores of normal, PanIN 1-2, and PanIN 3 pancreatic tissues in KC mice as described in panel **e** (*n* = 10 biologically independent samples). **g**, **h** Two independent iKPC cell lines were treated with 1 µg/ml doxycycline (DOX-on) or control solvent (DOX-off) for 72 h, and then PPARδ mRNA expression from three independent experiments was measured by qRT-PCR (**g**), and PPARδ and p-Erk1/2 protein levels were measured by Western blot (**h**). **i**, **j** Two iKPC mouse PDAC cell lines cultured with 1 µg/ml DOX were treated with 1 µM MEK inhibitor PD0325901 for 24 h, and then PPARδ mRNA expression from three independent experiments was measured by qRT-PCR (**i**), and PPARδ and p-Erk1/2 protein levels were measured by Western blot (**j**). Data are mean ± SEM. For (**b**, **f**), one-way ANOVA with Bonferroni correction, and for (**d**), (**g**, **i**), unpaired two-tailed Student’s *t*-test. *P < .05 and ****P < .0001. Source data are provided in a Source Data file.

To determine whether PPARδ is upregulated in the earlier stages of pancreatic tumorigenesis, we evaluated PPARδ mRNA expression by RNAscope in situ hybridization and protein expression by immunohistochemical (IHC) staining of PanINs in pancreata from patients and KC mice (*Pdx1-Cre;* LSL-*Kras*^*G12D*^), in which the *Kras*^*G12D*^ mutation (*KRAS*^*mu*^) is targeted to Pdx1^+^ pancreatic epithelial cells (Supplementary Fig. 1a). PPARδ expression was weak and limited to nuclei in normal human pancreatic tissues, but strong in the nuclei and cytoplasm of paired PanINs (grade 1-2) and further increased in PDAC (Fig. 1a, b, Supplementary Fig. 1b); PPARδ mRNA expression also increased in human PanIN 1-2 lesions compared to paired normal pancreatic tissues (Fig. 1c, d, Supplementary Fig. 1c, d). Similarly, in KC mice, PanINs had higher PPARδ expression than adjacent normal pancreatic tissues, and PPARδ expression was further increased with progression of PanINs (Fig. 1e, f, Supplementary Fig. 1e, f). Furthermore, the PPARδ upregulation was associated with a progressive increase of *KRAS*^*mu*^ activity (measured by p-Erk1/2 IHC), ductal metaplasia (Alcian blue staining for acidic mucins^28^), and pancreatic fibrosis (Sirius red staining for collagen fibrils^29^) (Fig. 1e, f, Supplementary Fig. 1g–i). Therefore, we next explored whether PPARδ expression was regulated by *KRAS*^*mu*^ activation. GEO2R analyses of publicly available mRNA profiling data (Gene Expression Omnibus database #GSE32277) revealed that *KRAS*^*mu*^ activation driven by doxycycline significantly increased PPARδ expression in five independent cultured doxycycline-inducible *KRAS*^*mu*^ p53-null mouse PDAC cell lines (iKPC)^30^ in vitro and in their pancreatic orthotopic xenograft tumors in vivo (Supplementary Fig. 1j, k). In our independent confirmatory experiments using two of the five iKPC cell lines^30^, doxycycline-induced *KRAS*^*mu*^ activation increased PPARδ mRNA and protein expression levels (Fig. 1g, h); and inhibition of *KRAS*^*mu*^ activity (measured by p-Erk1/2 Western blot) by a MEK inhibitor (PD0325901) significantly decreased PPARδ expression at both mRNA and protein levels (Fig. 1i, j). In those two iKPC cell lines, doxycycline induced GTP-bound active Kras, measured by a Raf pull-down assay, but interestingly and not surprisingly, PD0325901, which blocks the *KRAS*^*mu*^ signaling pathway (KRAS-RAF-MEK-ERK) downstream of Raf, increased active Kras levels possibly secondary to PD0325901 decreasing MEK negative regulatory feedback of Kras activity (Supplementary Fig. 1l). These data demonstrated that *KRAS*^*mu*^ activation upregulates PPARδ expression.

### PPARδ promotes *KRAS*^*mu*^*-*initiated pancreatic tumorigenesis specially when promoted by an HFD

**Fig. 2:**
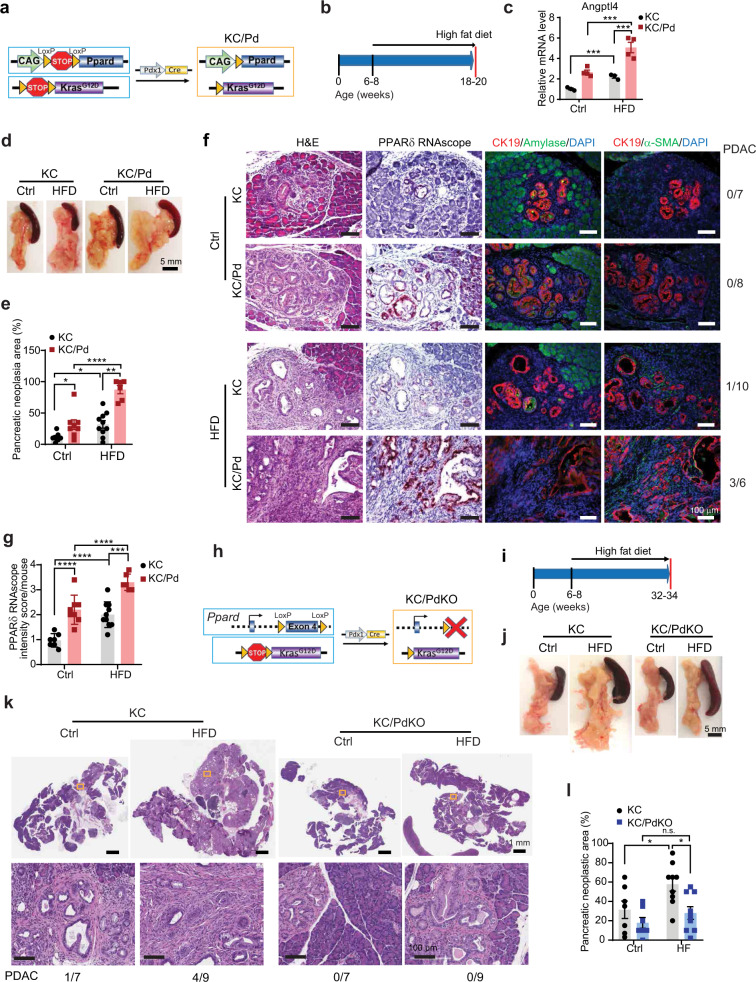
PPARδ promotes *KRAS*^*mu*^*-*initiated pancreatic tumorigenesis specially when augmented by a high-fat diet (HFD). **a** Schematic diagram of the generation of KC/Pd mice. **b**–**g** KC and KC/Pd mice at 6-8 weeks old, fed either the (high fat diet) HFD or the control diet (Ctrl) for 12 weeks, were euthanized, and pancreata were photographed, weighed, and harvested for gross and histologic characterization. **b** Schematic diagram for timeline of the HFD treatment. **c**–**f** mRNA relative expression of PPARδ target Angptl4 (**c**, *n* = 3-4 biologically independent samples); representative photos of pancreata (**d**); percentage of pancreatic neoplastic area per mouse (**e**, *n* = 6–10 per group); representative images of H&E staining, PPARδ RNAscope in situ hybridization, and co-immunofluorescence staining of CK19 with amylase or with α-SMA for the pancreata. PDAC incidence rates for each group are shown at the left side of each panel (**f**); quantitative results for PPARδ RNAscope in situ hybridization (**g**, *n* = 6–10 biologically independent samples) for the KC and KC/Pd mice on the HFD or Ctrl. **h** Schematic diagram of the generation of KC/PdKO mice. **i**–**l** KC and KC/PdKO mice at 6-8 weeks were fed either the HFD or Ctrl for 26 weeks. **i** Timeline for the HFD feeding of mice. Representative photos of pancreata (**j**); representative images of H&E staining of pancreata and PDAC incidence rates (bottom) (**k**); and the percentage of pancreatic neoplastic area per mouse (**l**, *n* = 7–9 per group) for KC and KC/PdKO mice on the HFD or Ctrl. Data are mean ± SEM. For (**c**), (**e**), (**g**), and (**l**), two-way ANOVA with Bonferroni correction. *P < .05, **P < .01, ***P < .001, and ****P < .0001. n.s.: no significance. Source data are provided in a Source Data file.

To investigate the mechanistic significance of PPARδ upregulation in PanIN-to-PDAC development, we generated a mouse model, Panc-Pd (Pdx1-*Cre*; CAG-LSL-*Ppard*), in which PPARδ overexpression is targeted to the pancreatic epithelial cells via Pdx1 promoter-driven Cre-recombinase expression (Supplementary Fig. 2a, b). Panc-Pd mice had higher pancreatic PPARδ protein expression levels than did their wild-type (WT) littermates (Supplementary Fig. 2c). Transgenic PPARδ overexpression in pancreatic epithelial cells without *KRAS*^*mu*^ was insufficient to induce pancreatic tumorigenesis in Panc-Pd mice (Supplementary Fig. 2d, e). However, this PPARδ overexpression in *KRAS*^*mu*^ pancreatic epithelial cells in KC/Pd mice (Pdx1-*Cre*; LSL-*Kras*^*G12D*^; CAG-LSL-*Ppard*) (Fig. 2a), generated by breeding of Panc-Pd mice with KC mice, significantly increased pancreatic neoplastic area compared with that of KC mice, with both groups aged 12-14 weeks or older (Supplementary Fig. 2d–f).

HFDs promote the progression of *KRAS*^*mu*^*-*initiated pancreatic tumorigenesis^11–18^ and are enriched with fatty acids that are natural ligands of PPARδ. We next examined whether fatty acids’ activation of PPARδ affects the HFD’s promotion of *KRAS*^*mu*^*-*initiated pancreatic tumorigenesis. KC and KC/Pd mice were fed either an HFD (60% kcal from fat) or a control diet (10% kcal from fat) for 12 weeks (Fig. 2b). The HFD increased pancreatic expression levels of PPARδ downstream target gene Angptl4, an indicator of PPARδ activity^31^, in KC mice, and this increase was greater in KC/Pd mice, confirming that PPARδ was hyperactivated by the HFD in KC/Pd mice (Fig. 2c). The HFD significantly increased pancreatic sizes and weights in KC mice and, to a greater extent, in KC/Pd mice (Fig. 2d, Supplementary Fig. 2g). More importantly, the HFD induced more pancreatic neoplastic lesion area, loss of pancreatic acinar cells (amylase positive), more metaplastic ductal cells (cytokeratin-19 [CK19] positive), and more fibrosis (α-smooth muscle actin [α-SMA]^+^) in KC mice and, to a greater extent, in KC/Pd mice (Fig. 2e, f). PDAC was observed in 10% (1/10) of KC mice and 50% (3/6) of KC/Pd mice fed the HFD for 12 weeks, but in none of the KC and KC/Pd mice fed the control diet for the same duration (Fig. 2f, right). Interestingly, the HFD increased not only PPARδ activity (Fig. 2c) but also PPARδ expression at both mRNA (Fig. 2f, g) and protein (Supplementary Fig. 2h, i) levels in both KC and KC/Pd mice. These results demonstrate that the PPARδ hyperactivation by the HFD strongly potentiates the progression of *KRAS*^*mu*^*-*initiated pancreatic carcinogenesis.

We subsequently sought to determine whether PPARδ in pancreatic epithelial cells is required for the HFD-induced promotion of *KRAS*^*mu*^*-*initiated pancreatic tumorigenesis. We therefore generated a mouse model with *Ppard* genetic deletion in *KRAS*^*mu*^ pancreatic epithelial cells by breeding KC mice with *Ppard* conditional knockout mice that have loxP sites flanking *Ppard* exon 4^32^ (KC/PdKO, Fig. 2h). The KC/PdKO mice had profound loss of PPARδ mRNA expression (Supplementary Fig. 2j). We next fed KC and KC/PdKO mice either the HFD or the control diet starting at age 6-8 weeks for 26 weeks (Fig. 2i). The HFD increased pancreatic sizes and weights and pancreatic neoplastic lesions in KC mice, but not in KC/PdKO littermates (Fig. 2j–l, Supplementary Fig. 2k). Furthermore, PDAC was observed in only KC mice (14% [1/7] on the control diet and 44% [4/9] on the HFD) and in none of the KC/PdKO mice fed either the control or the HFD (Fig. 2k). These findings demonstrated that PPARδ plays an essential role in the HFD-induced promotion of *KRAS*^*mu*^*-*initiated pancreatic tumorigenesis.

### Specific PPARδ activation by GW501516 strongly promotes *KRAS*^*mu*^*-*initiated pancreatic tumorigenesis

**Fig. 3:**
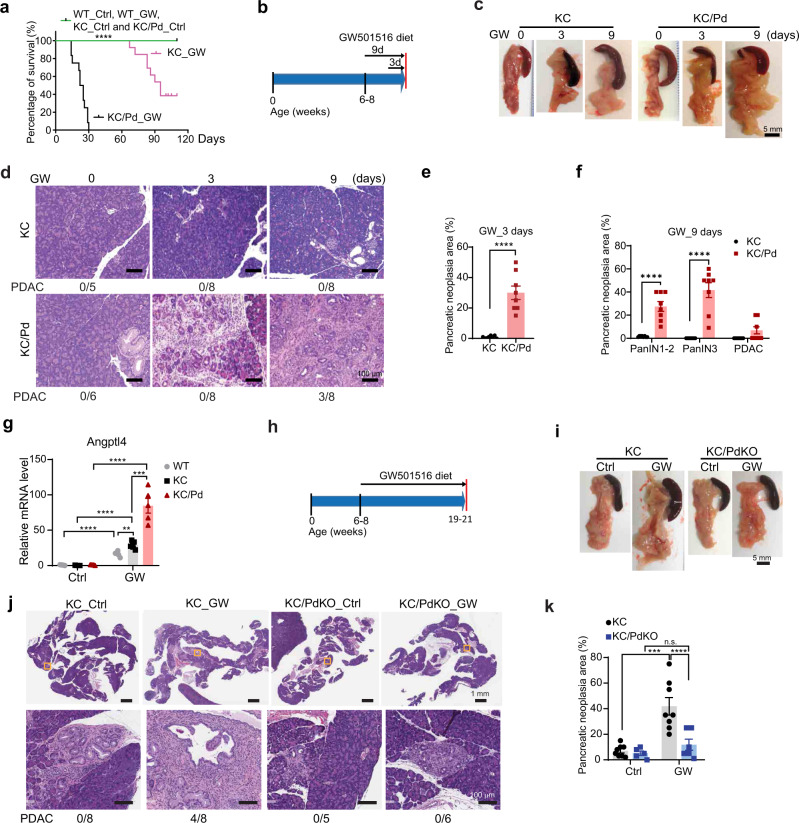
Specific PPARδ activation by GW501516 strongly promotes *KRAS*^*mu*^*-*initiated pancreatic tumorigenesis. **a** Survival of WT, KC, and KC/Pd mice on a GW501516 (GW) diet. WT, KC, or KC/Pd mice at age 6–8 weeks were fed a diet containing GW (50 mg/kg) or the same diet except without GW as a control (Ctrl) diet (*n* = 12–15 per group). The survival time was calculated by the Kaplan–Meier method and compared between groups using the log-rank test. ****P < .0001. **b**–**f** KC or KC/Pd mice at 6-8 weeks were fed the GW diet (50 mg/kg) for 0, 3, or 9 days and then euthanized (*n* = 5–8 per group). **b** Schematic diagram for timeline of GW diet feeding. **c**, **d** Representative photos of pancreata (**c**); and representative images of H&E staining of pancreata and incidence rates of PDAC/group (**d**) for KC and KC/Pd mice on the GW diet for 0, 3 and 9 days. **e** Percentage of pancreatic neoplastic area per mouse for KC and KC/Pd mice fed with the GW diet for 3 days (*n* = 8 per group). **f** Percentage of pancreatic neoplastic area per mouse for PanIN 1-2, PanIN 3, and PDAC for KC or KC/Pd mice on the GW diet for 9 days (*n* = 8 per group). **g** Angptl4 mRNA relative expression for WT, KC and KC/Pd mice on the GW or Ctrl diet for 9 days (*n* = 5 biologically independent samples). **h**–**k** KC and KC/PdKO mice at 6–8 weeks were fed either the GW (50 mg/kg) or the Ctrl diet for 13 weeks. **h** Timeline for the mice with the GW diet treatment. **i**–**k** Representative photos of pancreata (**i**); representative images of H&E staining of pancreata and incidence rates of PDAC (bottom) (**j**); and percentage of pancreatic neoplastic area per mouse (**k**, *n* = 5–8 per group) for KC and KC/PdKO on the GW or Ctrl diet. Data are mean ± SEM. For (**a**), log-rank test, for (**e**), unpaired two-tailed Student’s *t* test, for (**f**), multiple *t* tests, and for (**g**, **k**), two-way ANOVA with Bonferroni correction. *P < .05, **P < .01, ***P < .001, and ****P < .0001. n.s.: no significance. Source data are provided as a Source Data file.

HFDs contain various components in addition to fatty acids that might impact tumorigenesis. We therefore examined whether the observed effects of the HFD on *KRAS*^*mu*^*-*initiated pancreatic tumorigenesis were specifically related to PPARδ by using GW501516 (GW), a PPARδ-specific synthetic ligand^33^. KC and KC/Pd littermates were randomly assigned to be fed either a diet containing GW (50 mg/kg) or the same diet without GW (control) starting at age 6-8 weeks. While on the GW diet, all KC/Pd mice died within 30 days, and more than 50% of KC mice died by 90 days, but all of the sex- and age-matched KC or KC/Pd mice fed the control diet at the same time survived beyond 114 days (Fig. 3a). In addition, all of the sex- and age-matched WT mice with the same genetic background (C57BL/6) fed the same GW diet for 120 days survived well with no observed drug toxicity (e.g., loss of weight) (Fig. 3a). KC/Pd mice fed the GW diet for 14 days or more (e.g., 21 days) consistently showed large pancreas with a very hard consistency (Supplementary Fig. 3a), almost complete loss of acinar cells, severe pancreatitis, fibrosis, and advanced pancreatic neoplastic lesions; the same effects were seen to a much lesser extent in KC mice with the same treatment (Supplementary Fig. 3a, b). To further study the specific mechanistic significance of PPARδ in pancreatic tumorigenesis, we investigated the temporal development of pancreatic tumorigenesis by PPARδ hyperactivation via feeding the KC/Pd mice the GW diet for shorter time intervals of 3 days (control diet for 6 days and then GW diet for 3 days) and 9 days (GW diet for all 9 days) (Fig. 3b). While the KC mice showed only minimal PanINs after 9 days of GW treatment, all tested KC/Pd mice treated with GW for 3 days had significant acinar cell loss and developed pancreatitis and pancreatic neoplastic lesions, and these tumorigenic effects were intensified in KC/Pd mice on the GW diet for 9 days (Fig. 3c–f). Furthermore, PDAC was observed in 38% (3/8) of KC/Pd mice on the GW diet for 9 days (Fig. 3d). GW diet treatment for 9 days drastically increased Angptl4 expression in pancreata, but not in isolated splenic immune cells in KC/Pd mice, suggesting that GW specifically induced PPARδ hyperactivation in the pancreata to drive those tumorigenic effects (Supplementary Fig. 3c). GW also increased pancreatic Angptl4 expression in KC and WT mice, to a lesser extent (Fig. 3g), but did not affect their PPARδ expression levels (Supplementary Fig. 3d). GW treatment for 3 or 9 days also did not affect the body weights of KC and KC/Pd mice (Supplementary Fig. 3e). These data demonstrated that specific PPARδ hyperactivation by GW strongly promotes progression of *KRAS*^*mu*^*-*initiated pancreatic tumorigenesis.

To further characterize the specificity of PPARδ’s function in promoting the progression of *KRAS*^*mu*^*-*initiated pancreatic tumorigenesis, we examined the effects of *Ppard* KO in *KRAS*^*mu*^ pancreatic epithelial cells on the GW diet’s promotion of *KRAS*^*mu*^*-*initiated pancreatic tumorigenesis in KC/PdKO mice. Starting from age 6–8 weeks, KC or KC/PdKO mice were fed the GW or control diet (Fig. 3h). The GW diet failed to increase pancreatic weights and pancreatic neoplastic lesions, even after 13 weeks of GW treatment, in KC/PdKO mice (Supplementary Fig. 3f, Fig. 3i–k). In contrast, sex- and age-matched KC littermates on the same GW treatment for 13 weeks or less (some mice were euthanized earlier because of severe pancreatitis and tumorigenesis) had significantly higher pancreatic weights and larger pancreatic neoplastic lesions than did the KC mice on the control diet (Supplementary Fig. 3f, Fig. 3i–k). This GW treatment did not affect mouse body weight in either KC or KC/PdKO mice (Supplementary Fig. 3g). Furthermore, PDAC was observed in 50% (4/8) of the GW-fed KC mice, but in none of the KC mice fed the control diet or their KC/PdKO littermates fed either the GW diet or the control diet (Fig. 3j). These findings further demonstrated the specificity of PPARδ’s role in promoting the progression of *KRAS*^*mu*^*-*initiated pancreatic tumorigenesis.

### PPARδ hyperactivation promotes inflammation-related signaling pathways and *KRAS*^*mu*^ activity during pancreatic tumorigenesis

**Fig. 4:**
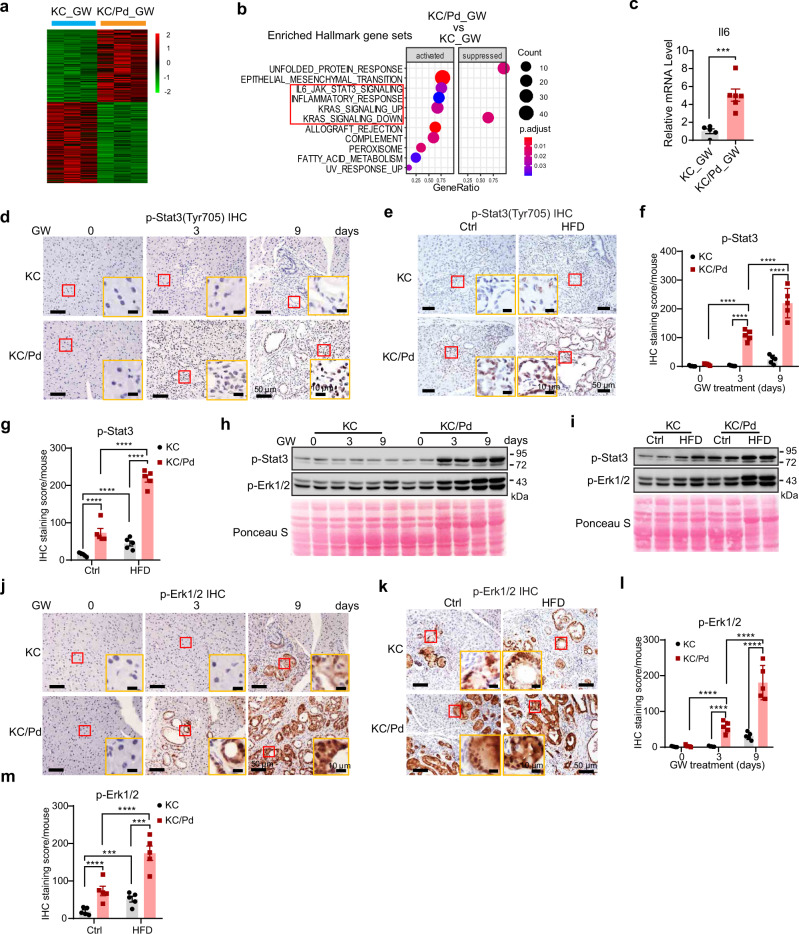
PPARδ promotes inflammation-related signaling pathways and *KRAS*^*mu*^ activation during pancreatic tumorigenesis. **a**, **b** KC and KC/Pd mice at 6−8 weeks of age were fed the GW diet (50 mg/kg) for 3 days. The total RNA of pancreata from these mice was extracted for RNA-sequencing transcriptome profile analyses (*n* = 3−4 per group). **a** Heatmap of differentially expressed genes with *P*(Adj)<0.05 between the GW diet–treated KC (KC_GW) and KC/Pd (KC/Pd_GW) mouse groups. **b** Pathway enrichment results for KC/Pd_GW vs. KC_GW mice obtained by gene set enrichment analyses using R package “ClusterProfiler”, a cutoff of *P*(Adj)=0.05, and gene sets=MSigDB category “Hallmark gene sets”. The inflammation-related and KRAS signaling pathways are marked in a red square. **c** Il6 mRNA levels measured by qRT-PCR of pancreata from the KC and KC/Pd mice at 6–8 weeks of age fed the GW diet for 3 days, (*n* = 5–6 biologically independent samples). **d**–**m** Representative images of IHC staining for p-Stat3 (Tyr705) (**d**, **e**) and their quantitative results (**f**, **g**, *n* = 5 biologically independent samples), Western blot for p-Stat3 and p-Erk1/2 (**h**, **i**), and IHC staining for p-Erk1/2 (**j**, **k**) and their quantitative results (**l**, **m**, *n* = 5 biologically independent samples) for KC and KC/Pd mice on the GW diet for 0, 3, and 9 days as described in Fig. 3b (**d**, **f**, **h**, **j**, **l**) or on the HFD or Ctrl for 12 weeks as described in Fig. 2b (**e**, **g**, **i**, **k**, and **m**). Data are mean ± SEM. For (**c**), unpaired two-tailed Student’s *t* test, and for (**f**), (**g**), (**l**, **m**), two-way ANOVA with Bonferroni correction. ***P < .001, and ****P < .0001. Source data are provided as a Source Data file.

To identify the mechanisms by which PPARδ hyperactivation promoted pancreatic tumorigenesis (Fig. 2), we performed comparative RNA sequencing (RNA-seq) for transcriptome profiling studies of pancreata from KC and KC/Pd mice fed the GW diet for 3 days. Pancreata of GW-treated KC/Pd and KC mice had distinctive differential expression patterns with 1498 differentially expressed genes according to a cutoff of *P*(Adj)<.05, including 709 upregulated genes and 789 downregulated genes (Fig. 4a, Supplementary Fig. 4a). Gene set enrichment analyses of these 1498 differentially expressed genes using the “Hallmark gene sets” category showed that the top enriched pathways included IL6-JAK-STAT3, inflammatory response, and KRAS signaling (Fig. 4b, Supplementary Fig. 4b, c).

Stat3 activation by Il6 plays an essential role in promoting *KRAS*^*mu*^*-*initiated pancreatic tumorigenesis^34,35^. *KRAS*^*mu*^ activation by inflammatory mediators (e.g., Il6) strongly promotes the progression of pancreatic tumorigenesis in KC mice^36,37^. With 3 days of GW treatment, KC/Pd mice had higher Il6 mRNA expression than did KC mice (Fig. 4c). PPARδ hyperactivation by the GW diet or the HFD significantly increased expression levels of p-Stat3 (Tyr705) (Fig. 4d–i) and *KRAS*^*mu*^ activity (p-Erk1/2) (Fig. 4h–m) measured by IHC and Western blot in KC mice and, to a greater extent in KC/Pd mice. STAT3 activation is characterized by Tyr705 and Ser727 phosphorylation, and Tyr705 is generally regarded as the main actuator for the IL6/STAT3 signaling pathway^34^.

To further explore the mechanisms by which PPARδ increased *KRAS*^*mu*^ activity to promote progression of pancreatic tumorigenesis, we performed in vitro studies using PPARδ overexpression via lentivirus transduction or GW treatment of a panel of human and mouse PDAC cells. We found that neither PPARδ overexpression (Supplementary Fig. 4d, e) nor PPARδ activation by GW treatment (Supplementary Fig. 4f, g) increased p-ERK1/2 (Supplementary Fig. 4d, e, h, i) in the tested mouse and human PDAC cell lines, indicating that this in vivo PPARδ hyperactivation upregulated Il6/p-Stat3 and amplified *KRAS*^*mu*^ activity via indirect mechanisms, likely through remodeling the pancreatic tumor microenvironment (TME), rather than direct autochthonous effects.

### PPARδ hyperactivation by GW or HFD recruits macrophages and myeloid-derived suppressor cells (MDSCs) into the pancreata to remodel the immune TME

**Fig. 5:**
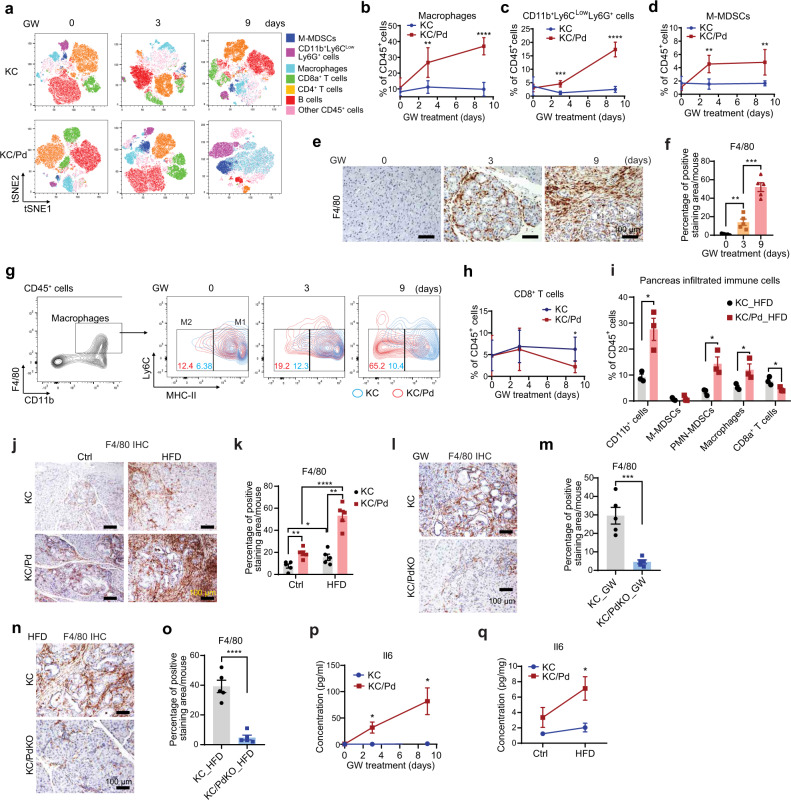
PPARδ activation by GW or HFD recruits macrophages and MDSCs into the pancreata to remodel pancreatic TME. **a** Representative immune cell profiling by FlowJo-tSNE (t-distributed stochastic neighbor embedding) of pancreas-infiltrating CD45^+^ cells, including M-MDSCs (CD11b^+^Ly6C^hi^Ly6G^−^), CD11b^+^Ly6C^low^Ly6G^+^ [polymorphonuclear (PMN)-MDSCs and neutrophil cells]), macrophages (CD11b^+^F4/80^+^), CD3^+^CD4^+^ cells, CD3^+^CD8^+^ T cells, and B cells in KC and KC/Pd mice fed the GW diet (50 mg/kg). **b**–**d** Quantitative results for macrophages (**b**), CD11b^+^Ly6C^low^Ly6G^+^ cells (**c**), and M-MDSCs (**d**) for the indicated mouse groups (*n* = 5–6). **e**, **f** Representative IHC staining images of macrophages (F4/80^+^) (**e**) and their quantitative results (**f**) for KC/Pd mice on the GW diet (*n* = 5). **g** Representative flow cytometry images and quantitative results of the pancreas-infiltrating Ly6C^low^ inflammatory macrophages with low MHC-II expression (M2-TAMs). **h** Quantitative results of CD8^+^ T cells (*n* = 5–6). **i**–**k** Quantitative results of the indicated immune cells in pancreas-infiltrating CD45^+^ cells (**i**, *n* = 3), and representative IHC staining images of macrophages (F4/80^+^) (**j**) and their quantitative results (**k**) in pancreata for KC and KC/Pd mice fed the HFD or Ctrl for 12 weeks (*n* = 5). **l**, **m** Representative IHC staining images of macrophages (F4/80^+^) (**l**) and their quantitative results (**m**) in pancreata for KC and KC/PdKO mice fed the GW for 13 weeks (*n* = 5). **n**, **o** Representative IHC staining images of macrophages (F4/80^+^) (**n**) and their quantitative results (**o**) in pancreata for KC and KC/PdKO mice fed HFD for 26 weeks (*n* = 5). **p**, **q** Il6 protein expression levels in the pancreata of KC and KC/Pd on the GW diet for 0, 3, or 9 days (**p**, *n* = 3–4) or on the HFD or Ctrl for 12 weeks (**q**, *n* = 3-4). For (**b**–**d**), (**h**), data are mean ± SD, and for (**f**), (**I**), (**k**), (**m**), (**o**–**q**), data are mean ± SEM. For (**b**–**d**), (**h**), (**I**), (**p**), (**q**), multiple *t*-tests, for (**f**), one-way ANOVA with Bonferroni correction, for (**k**), two-way ANOVA with Bonferroni correction, and for (**m**, **o**), unpaired two-tailed Student’s *t* test. *P < .05, **P < .01, ***P < .001, and ****P < .0001, compared to KC mice with the same diet treatment for (**b**–**d**), (**h**), (**p,** **q**). Source data are provided as a Source Data file.

Based on our findings showing that PPARδ hyperactivation promoted inflammation-related signaling pathways (e.g., Il6/Stat3) in mouse pancreata (Fig. 4b–i and Supplementary Fig. 4b, c), we further investigated the role of PPARδ in regulating pancreatic immune and inflammatory responses to promote pancreatic tumorigenesis by profiling immune/inflammatory cells via flow cytometry in the pancreata of KC and KC/Pd mice fed the GW diet for 3 and 9 days, or the HFD for 12 weeks, as described before (Fig. 3b for GW and Fig. 2b for HFD). The GW treatment for a short time did not change accumulations of pancreatic immune cells in KC mice (Fig. 5a–d). However, the same GW treatment increased the accumulation of pancreatic myeloid cells (CD11b^+^), including macrophages (CD45^+^CD11b^+^F4/80^+^), polymorphonuclear (PMN) MDSCs, neutrophils (CD45^+^CD11b^+^Ly6C^low^Ly6G^+^), and mononuclear (M) MDSCs (CD45^+^CD11b^+^Ly6C^hi^Ly6G^−^) in KC/Pd mice compared to the KC littermates at 3 and 9 days of GW treatment (Fig. 5a–f, Supplementary Fig. 5a, b). Of note, while PMN-MDSCs and neutrophils share many morphological and phenotypic features, PMN-MDSCs differentially express CD14^38^. Among macrophages, which were the dominant immune cells infiltrating into the pancreatic TME, the proportion of M2 tumor-associated macrophages (M2-TAMs; CD45^+^CD11b^+^F4/80^+^Ly6C^low^MHC-II^low^)^39,40^ increased from 12.4% in KC/Pd mice fed the control diet (GW for 0 days) to 19.2% and 65.2% in KC/Pd mice fed the GW diet for 3 and 9 days, respectively; GW also increased M2-TAMs in KC mice, although to a lesser degree (Fig. 5g). Among MDSCs, PMN-MDSCs (CD45^+^CD11b^+^Ly6C^low^Ly6G^+^CD14^+^)^38^ were increased at 3 days and further increased at 9 days of GW treatment (Supplementary Fig. 5c, d) while the increase in M-MDSCs plateaued after 3 days of GW treatment (Fig. 5d) in KC/Pd mice. In contrast, GW significantly decreased levels of CD8^+^ T cells (Fig. 5h) and CD4^+^ T cells (Supplementary Fig. 5e) in KC/Pd mice compared to the KC littermates at 9 days of treatment, and KC/Pd mice had lower B-cell accumulation than KC mice did at 9 days of GW treatment, but this trend failed to reach statistical significance (Supplementary Fig. 5f). Pancreata of KC/Pd mice on the HFD also had significantly higher accumulation of CD45^+^CD11b^+^ cells, macrophages, and MDSCs but lower accumulation of CD8^+^ T cells than the pancreata of KC mice on the HFD did (Fig. 5i). To further confirm the results from flow cytometry, we performed IHC staining for F4/80 and immunofluorescence staining for Gr1 on the paraffin-embedded pancreatic tissues from the same HFD experiment and found that HFD increased accumulations of pancreatic macrophages (Fig. 5j, k) and MDSCs (Supplementary Fig. 5g, h) in KC mice and, to a greater extent, in KC/Pd mice compared to control diet. In contrast, *Ppard* genetic deletion in pancreatic epithelial cells in KC/PdKO mice significantly reversed the effects promoted by GW treatment (Supplementary Fig. 6a-d) and the HFD (Supplementary Fig. 6e, f) in KC mice by decreasing macrophages (Fig. 5l–o) and decreasing MDSCs (Gr1^+^) (Supplementary Fig. 6c–f).

Next, we screened for cytokines that might mediate proinflammatory effects by PPARδ activation in pancreata of KC and KC/Pd mice fed the GW diet for 3 or 9 days or the HFD for 12 weeks using a mouse proinflammatory cytokine panel of 13 major inflammation-related cytokines (BioLegend)^41^. The screen results revealed increases in 5 of 13 screened cytokines (Il6, Tnfa, Csf2, Il1a, and Ccl2) at 3 days and 9 days of the GW treatment in KC/Pd mice compared to KC mice on the same treatment or to KC/Pd mice on the control diet (GW for 0 days) (Fig. 5p, Supplementary Fig. 6g–k). Similarly, we observed the increases in Il6, Tnfa, and Ccl2 in KC/Pd mice on the HFD compared to KC mice on the same treatment or to KC/Pd mice on the control diet (Fig. 5q, Supplementary Fig. 6l–n).

### PPARδ alteration modulates chemokine CCL2 expression in mouse and human pancreatic epithelial cells in vivo and in vitro

**Fig. 6:**
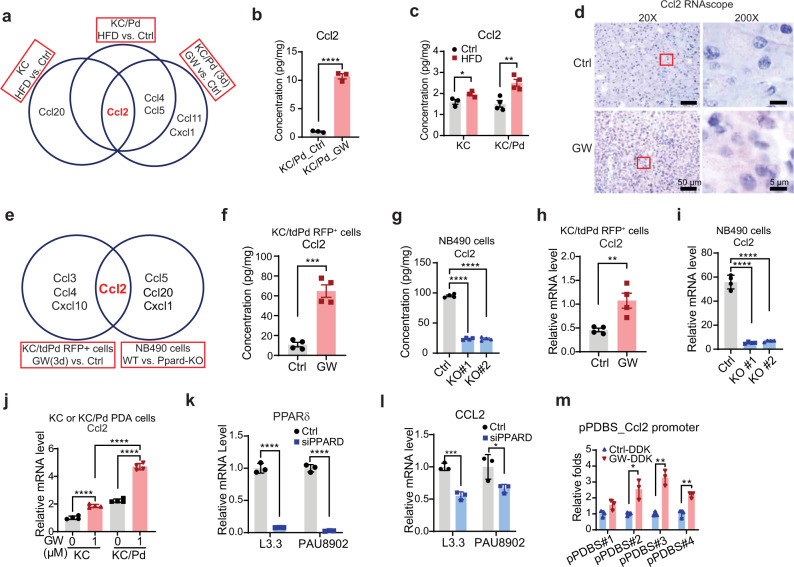
PPARδ alteration directly modulates chemokine CCL2 expression in mouse and human pancreatic epithelial cells. **a**–**c** Pancreatic tissues from KC or KC/Pd mice fed the HFD or Ctrl diet; and KC/Pd mice fed the GW or Ctrl diet for 3 days (*n* = 3–4 biologically independent samples). Venn diagram of differentially expressed chemokines (**a**). Quantitative results of Ccl2 protein levels for KC/Pd mice on the GW diet (**b**) and KC or KC/Pd mice on the HFD (**c**). **d** Representative images of Ccl2 RNAscope in situ hybridization for normal pancreatic tissues of KC/Pd mice fed the GW or Ctrl diet for 3 days (*n* = 3 biologically independent samples). **e** Venn diagram of differentially expressed chemokines for mouse tdTomato RFP–sorted pancreatic epithelial cells of KC/tdPd mice on GW or control diet for 3 days and for the cell culture media from mouse NB490 KPC cells with WT or Ppard KO. **f**, **g** Quantitative results of Ccl2 protein levels for tdTomato-RFP^+^ cells (**f**, *n* = 4 biologically independent samples) and for the cell culture media from mouse NB490 KPC cells from 4 independent experiments (**g**). **h**, **i** Ccl2 mRNA expression levels in tdTomato-RFP^+^ pancreatic epithelial cells (**h**, *n* = 4 biologically independent samples) and in mouse NB490 KPC cells from 4 independent experiments (**i**). **j** Ccl2 mRNA expression levels in mouse KC and KC/Pd PDAC cells with or without GW treatment from 4 independent experiments. **k**, **l** PPARδ (**k**) and CCL2 (**l**) mRNA expression levels in human PDAC cells transfected with PPARδ siRNAs (siPPARD) or control siRNA (Ctrl) from 3 independent experiments. **m** The PPARδ binding to the four predicted PPARδ binding sites (pPDBS) in the mCcl2 promoter in mouse KC PDAC cells stably transduced with mouse DDK-tagged PPARδ expressing lentivirus and treated with 1 µM GW or solvent (DMSO) from 3 independent experiments. Data are mean ± SEM. For (**b**), (**f**), (**h**), unpaired two-tailed Student’s *t* test, for (**c**), (**k**–**m**), multiple *t*-tests, for (**g**), (**i**), one-way ANOVA with Bonferroni correction, and for (**j**), two-way ANOVA with Bonferroni correction. *P < .05, **P < .01, ***P < .001, and ****P < .0001. Source data are provided as a Source Data file.

To further investigate the mechanisms by which PPARδ hyperactivation increased the recruitment of inflammatory and immunosuppressive cells such as macrophages and MDSCs into the TME of pancreata, we screened for a panel of chemokines in pancreatic tissues of KC and KC/Pd mice fed the GW diet for 3 and 9 days or fed the HFD for 12 weeks using the LEGENDplex Mouse Proinflammatory Chemokine Panel, which simultaneously quantifies 13 major inflammatory chemokines^41^. KC/Pd mice fed the GW diet for 3 and 9 days had significantly higher production of 5 chemokines (Ccl2, Ccl4-5, Ccl11, and Cxcl1) and 8 chemokines (Ccl2-4, Ccl11, Ccl17, Ccl20, Cxcl1, and Cxcl5), respectively, compared to KC/Pd mice on the control diet (GW diet for 0 days) or to KC mice on the same treatment (Fig. 6a, b, Supplementary Fig. 7a–i). Similarly, KC/Pd mice fed the HFD had significantly higher production of 3 chemokines (Ccl2 and Ccl4-5) than did KC/Pd mice on the control diet, while KC mice fed the HFD had higher Ccl2 and Ccl20 production than did KC mice on the control diet (Fig. 6a, c, Supplementary Fig. 7j–l). Our screening results for pancreatic tissues indicated that although PPARδ hyperactivation regulated various chemokines, Ccl2 was the only common upregulated chemokine across three settings: 1) the GW diet compared with the control diet for 3 days of treatment in KC/Pd mice; 2) the HFD compared with the control diet in KC/Pd mice, and 3) the HFD compared with the control diet in KC mice (Fig. 6a–c, Supplementary Fig. 7a–l).

We then evaluated whether PPARδ hyperactivation modulated Ccl2 in pancreatic epithelial cells. We first performed Ccl2 RNAscope in situ hybridization to localize Ccl2 mRNA expression in pancreatic cell subpopulations and found that PPARδ hyperactivation by GW treatment for 3 days in KC/Pd mice significantly increased Ccl2 mRNA expression in normal pancreatic epithelial cells compared to the same mice on the control diet (Fig. 6d). In contrast, *Ppard* KO in KC mice significantly decreased Ccl2 mRNA expression and reversed GW-upregulated and HFD-upregulated Ccl2 mRNA expression (Supplementary Fig. 7m–p). Furthermore, we sorted by flow cytometry enzymatically digested tdTomato red fluorescence protein (RFP)-marked pancreatic epithelial cells from the pancreatic tissues of KC/tdPd mice (Supplementary Fig. 8a), which were generated as described in the Methods section and fed either the GW diet or the control diet for 3 days (Supplementary Fig. 8b–d). tdTomato-RFP–sorted cells were characterized prior to chemokine profiling using the LEGENDplex Mouse Proinflammatory Chemokine Panel. The GW treatment increased CK19 but decreased amylase mRNA expression levels (Supplementary Fig. 8e, f) while increasing 4 of 13 chemokines (Ccl2-4 and Cxcl10) in tdTomato-RFP–sorted pancreatic epithelial cells of KC/tdPd mice (Supplementary Fig. 8g–j). In addition, we used the same method to screen the differentially expressed chemokines in the cell culture media of mouse NB490 KPC cells^42^ with WT *Ppard* or with *Ppard* KO by the CRISPR method as described in the Methods section (Supplementary Fig. 8k) and found that protein levels of 4 of the 13 chemokines (Ccl2, Ccl5, Ccl20, and Cxcl1) were decreased by *Ppard* KO in the culture media (Fig. 6e, Supplementary Fig. 8l). Thus, the chemokine screening results for pancreatic epithelial cells also showed that Ccl2 was the only common chemokine regulated by multiple methods of PPARδ alterations (Fig. 6e–g, Supplementary Fig. 8g–l), which was further confirmed by another set of independent experiments assessing these cells’ Ccl2 mRNA expression levels (Fig. 6h, i). Similar results for Ccl2 mRNA expression were also observed in 1) PDAC cell lines generated from pancreatic tumors of KC or KC/Pd mice that were treated with GW or its solvent control (Fig. 6j); 2) KC PDAC cells transduced with control or mouse PPARδ expressing lentivirus and further treated with GW (Supplementary Fig. 8m); and 3) two mouse iKPC under doxycycline challenge treated with GW or its solvent control (Supplementary Fig. 8n).

We next evaluated the translational relevance of our mouse findings in experiments of human PDAC cells. In two different human PDAC cell lines, PPARδ downregulation by PPARδ siRNAs decreased CCL2 mRNA expression (Fig. 6k, l) but did not significantly change p-STAT3 (Tyr705) expression (Supplementary Fig. 9a). In addition, PPARδ overexpression by PPARδ-expressing lentivirus increased CCL2 mRNA expression in two human PDAC cell lines and GW treatment increased CCL2 mRNA expression in 3 of 6 human PDAC cells (Supplementary Fig. 9b, c), likely reflecting the heterogeneity of established human PDAC cells, which could be secondary to in vitro acquired alterations. Oncomine public database analyses showed that PPARδ mRNA expression levels were upregulated in human PDACs compared to adjacent normal pancreatic tissues in the Badea pancreas set, and this PPARδ upregulation occurred alongside significant upregulation of several chemokines such as CCL2-5, CXCL1, and CXCL10 and cytokines such as IL6 (Supplementary Fig. 9d), which demonstrates the clinical relevance of our mouse findings. Another analysis from the Pan-Cancer Atlas public database, using TCGA data, also showed that PPARδ expression was positively correlated with CCL2 expression (Supplementary Fig. 9e). Furthermore, a ChIP-qPCR assay showed that GW treatment increased PPARδ binding to 3 of 4 predicted PPARδ binding sites in the murine Ccl2 promoter in murine KC PDAC cells stably transduced with a DDK-tagged PPARδ-expressing lentivirus (Supplementary Fig. 9f, Fig. 6m) and murine KC PDAC cells (Supplementary Fig. 9g), and also increased PPARδ binding to the predicted PPARδ binding site in the human CCL2 promoter of Panc-1 PDAC cells (Supplementary Fig. 9h, i). All these findings demonstrate that PPARδ directly regulates CCL2 expression in both mouse and human pancreatic epithelial cells.

### PPARδ hyperactivation upregulates Ccl2/Ccr2 signaling to promote pancreatic tumorigenesis

**Fig. 7:**
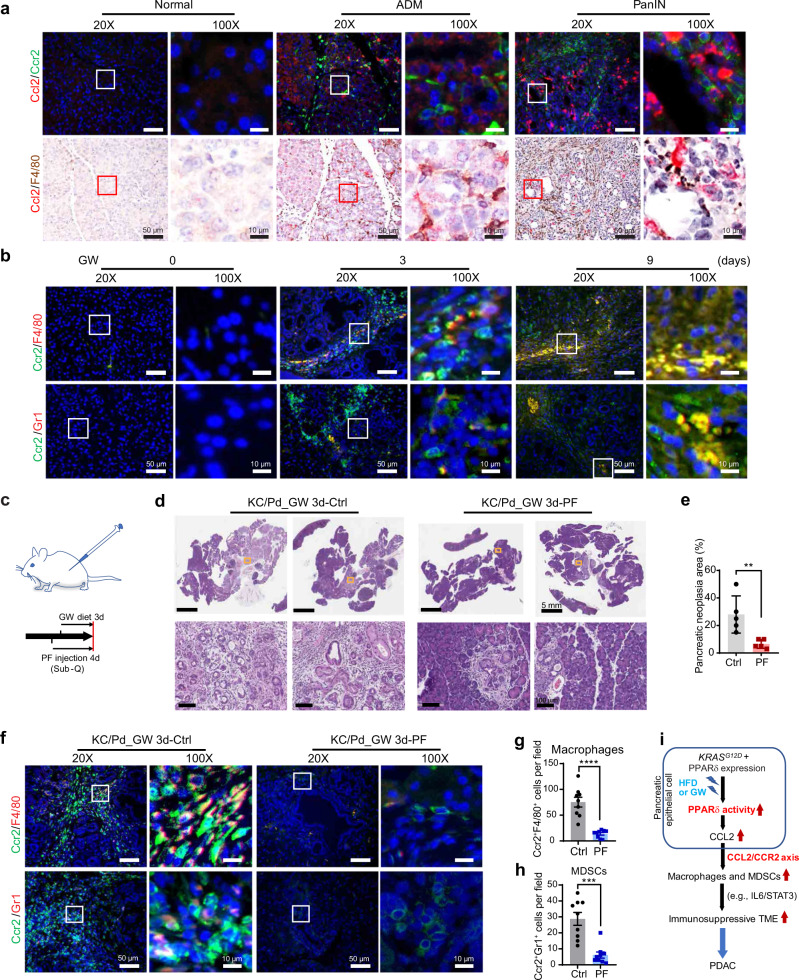
Pharmacological inhibition of Ccr2 suppresses pancreatic tumorigenesis promoted by PPARδ-Ccl2/Ccr2-TAMs/MDSCs. **a** Representative image of co-staining for Ccl2 RNAscope in situ hybridization with Ccr2 IF (top) or with F4/80 IHC (bottom) in pancreatic normal, acinar-to-ductal metaplasia (ADM), and PanIN tissues in the KC/Pd mice fed the GW diet for 3 days (*n* = 5 per group). **b** KC/Pd mice at 6–8 weeks on the GW diet (50 mg/kg) for 0, 3, or 9 days were euthanized, and then pancreatic tissues were harvested for further analyses (*n* = 4–6 per group). Representative images of co-IF staining of Ccr2 with F4/80 (TAMs) (top) or with Gr1 (MDSCs) (bottom). **c**–**h** Ccr2 inhibitor PF4136309 (PF) or control solvent (corn oil) was administered via subcutaneous injection at 80 mg/kg twice daily to the KC/Pd mice for 4 days (*n* = 5 mice per group), and the mice were fed the GW diet (50 mg/kg) for the last 3 days, and then pancreatic tissues were harvested for further analyses. **c** Timeline for the mice with PF4136309 and GW diet treatment. **d**, **e** Representative images of H&E staining of pancreata (**d**) and percentage of pancreatic neoplastic area per mouse (**e**) for GW-fed KC/Pd mice treated with PF4136309 or Ctrl. **f**–**h** Representative images of co-IF staining of Ccr2 with F4/80 (TAMs) (**f**, top) or with Gr1 (MDSCs) (**f**, bottom), and quantitative co-IF staining results of double-positive cells per 40× field for Ccr2^+^/F4/80^+^ cells (**g**) and Ccr2^+^/Gr1^+^ cells (**h**) for the indicated mice. **i** Schematic flow showing PPARδ hyperactivation by either the HFD or the GW diet upregulates CCL2, which chemoattracts macrophages and MDSCs into pancreata via the CCL2/CCR2 axis, leading to an inflammatory and immunosuppressive TME (e.g., IL6/STAT3) and subsequent progression of *KRAS*^*mu*^-initiated pancreatic tumorigenesis to PDAC, while *PPARD*-genetic KO and a CCR2 inhibitor (PF4136309) suppress those tumorigenic effects. Data are mean ± SEM. Unpaired two-tailed Student’s *t* test. **P < .01, ***P < .001, and ****P < .0001. Source data are provided as a Source Data file.

On the basis of our findings that macrophages were the most infiltrating immunosuppressive inflammatory cells in pancreata of KC/Pd mice with PPARδ hyperactivation (Fig. 5) and that CCL2 expression was directly regulated by *PPARD* alterations in human and mouse pancreatic epithelial cells (Figs. 6, 7), we examined whether PPARδ upregulation of Ccl2 secretion by pancreatic epithelial cells increased Ccr2^+^ immune cells in the pancreata of KC/Pd mice fed the GW diet, given that Ccr2 is the primary Ccl2 receptor and commonly present in immune cells such as macrophages and MDSCs^43,44^. GW-induced PPARδ hyperactivation significantly increased Ccl2 production in pancreatic epithelial cells and infiltration of Ccr2^+^ immune cells, especially macrophages, into pancreatic neoplastic lesions at early stages, as shown in acinar-to-ductal metaplasia (ADM) and PanINs, compared to their paired normal areas in the same pancreatic tissues (Fig. 7a). This result suggested that PPARδ-Ccl2/Ccr2-TAMs signaling significantly contributed to the progression of *KRAS*^*mu*^*-*initiated pancreatic tumorigenesis in this model. GW increased the proportions of Ccr2^+^ F4/80^+^ cells (TAMs) and Ccr2^+^ MDSCs in pancreata of KC/Pd mice following 3 days of treatment, and these levels were further increased at 9 days of treatment (Fig. 7b, Supplementary Fig. 10a–c). Early-stage pancreatic neoplastic lesions such as PanINs in KC/Pd mice fed GW had higher levels of infiltrating Ccr2^+^ TAMs than Ccr2^+^ MDSCs in the pancreatic TME at the earlier time point of 3 days of treatment (Fig. 7b, Supplementary Fig. 10a–c). Ccl2 increases macrophage migration, and inhibition of Ccr2 by a CCR2 inhibitor blocks macrophage migration in conditioned media from iKPC cells^45^. The conditioned media from pancreatic organoids derived from GW diet–treated KC/tdPd mice (described in Fig. 6e) significantly increased the migration of MDSCs (CD11b^+^Gr1^+^), which were sorted from the spleens of C57BL/6 WT mice by flow cytometry with fluorescence-labeled CD11b and Gr1 antibodies, compared to the pancreatic organoid media from the same mice on the control diet (Supplementary Fig. 10d–f).

Next, we examined the mechanistic significance of the Ccl2/Ccr2 axis in PPARδ hyperactivation–recruited macrophages and MDSCs in the pancreatic TME to promote progression of pancreatic tumorigenesis by subcutaneous administration^46^ of the CCR2 inhibitor PF4136309^47^ into KC/Pd mice fed the GW diet (Fig. 7c). CCR2 inhibitor treatment significantly suppressed GW-promoted pancreatic tumorigenesis, as shown by decreasing pancreatic neoplastic lesion area (Fig. 7d, e) and decreasing pancreatic infiltration with Ccr2^+^ TAMs and Ccr2^+^ MDSCs (Fig. 7f–h). These results strongly support the concept that PPARδ upregulation of CCL2 expression triggers recruitment of TAMs and MDSCs via the CCL2/CCR2 axis to foster an immunosuppressive TME and thus to promote the progression of *KRAS*^*mu*^*-*initiated pancreatic tumorigenesis (Fig. 7i).

## Discussion

In the present study, we found: (1) PPARδ expression was upregulated in precancerous PanINs in humans and KC mice, and the increase in *KRAS*^*mu*^ activity with PanIN progression positively upregulated PPARδ. (2) PPARδ activation, by its natural ligands such as fatty acids in an HFD or by its highly specific synthetic ligand GW501516, promoted pancreatic tumorigenesis, especially with pancreatic PPARδ upregulation, as experimentally modeled in KC/Pd mice. (3) *Ppard* genetic KO in pancreatic epithelial cells suppressed the pancreatic tumorigenesis promoted by HFD and GW in KC/PdKO mice. (4) PPARδ hyperactivation in pancreatic epithelial cells increased production of chemokine Ccl2 to recruit macrophages and MDSCs into pancreata via Ccl2/Ccr2 signaling, establishing an inflammatory and immunosuppressive TME to accelerate progression of pancreatic tumorigenesis. (5) Pharmacological inhibition of CCR2 suppressed pancreatic carcinogenesis promoted by PPARδ-mediated Ccl2/Ccr2-TAMs/MDSCs signaling in KC/Pd mice. These findings demonstrated that activation of upregulated PPARδ in PanINs can strongly promote progression of asymptomatic PanIN to PDAC.

Although it has been reported that PPARδ is upregulated in the late stage of pancreatic tumorigenesis in PDAC^22,25^, in this study, we demonstrate that PPARδ is also upregulated in early stages of human and mouse pancreatic tumorigenesis such as PanINs. Our findings also demonstrate that this PPARδ upregulation in PanINs is driven by *KRAS*^*mu*^ activation. In prior reports, *KRAS*^*G12V*^ signaling positively modulated PPARδ expression in transformed rat intestinal epithelial cells in in vitro studies^48^. A later comparative study of APC^Min/+^ mice with and without *KRAS*^*G12D*^ however showed no difference in PPARδ intestinal expression^49^. While different KRAS mutations might have different effects on PPARδ expression, our results show that activation of *KRAS*^*G12D*^, the most common KRAS mutation in pancreatic cancer, upregulates PPARδ expression during early pancreatic tumorigenesis not only in in vitro studies, but also, more importantly, in in vivo studies.

*KRAS* mutations are widely considered “constitutively active”; however, many healthy people possess PanINs harboring *KRAS* mutations without apparent harmful effects; in animals, *KRAS*^*mu*^ causes pancreatic neoplastic lesions only after *KRAS*^*mu*^ activation is increased by external factors^36,37^. Thus, the progression of *KRAS*^*mu*^-initiated pancreatic tumorigenesis requires additional factors (e.g., chronic pancreatitis, obesogenic HFD) to enhance *KRAS*^*mu*^ activation and modulate TME^12,16^. Our results demonstrate the important role of PPARδ in promoting the progression of *KRAS*^*mu*^-initiated tumorigenesis. For example, although an HFD is known to promote *KRAS*^*mu*^*-*initiated pancreatic tumorigenesis^7^, our results identify PPARδ hyperactivation as a critical mechanism for the HFD’s pro-tumorigenic effects. PPARδ’s mechanistic role in promoting pancreatic tumorigenesis is not limited to the HFD, as we have demonstrated using GW501516, a specific, synthetic PPARδ ligand^33^, which caused drastic acceleration of pancreatic tumorigenesis in KC/Pd mice, leading to PDAC development within 9 days and death of these mice within 30 days after GW treatment. This drastic acceleration of PDAC development demonstrated the potential powerful effects of PPARδ hyperactivation on progression of *KRAS*^*mu*^-initiated PDAC carcinogenesis. In a complementary *Ppard* genetic KO mouse model, PPARδ loss of expression in pancreatic epithelial cells also strongly suppressed the pancreatic carcinogenesis promoted by the HFD or the GW diet in KC/PdKO mice, further confirming the significance of PPARδ’s mechanistic role in promoting the progression of pancreatic carcinogenesis. These findings have important potential for clinical/translational implications: PPARδ activation by intake of PPARδ ligands, whether synthetic (e.g., GW) or natural (e.g., high levels of fatty acids in Western diets), can be potentially harmful for individuals whose pancreata harbor *KRAS*^*mu*^ PanINs, a scenario that increases with age in humans^50^.

PPARδ promotion of carcinogenesis via chronic inflammatory mechanisms has been described for colorectal and gastric cancer^41,51,52^. Our findings, however, demonstrate not only the applicability of this role to pancreatic carcinogenesis but also, more importantly, how PPARδ hyperactivation modulates the TME by recruitment of immunosuppressive cells (e.g., M2-TAMs and MDSCs) into the pancreatic TME, leading to decreased pancreatic infiltration of CD8^+^ effector T cells. M2-TAMs and MDSCs have emerged as important factors for the progression of *KRAS*^*mu*^*-*initiated pancreatic tumorigenesis by facilitating tumor escape of immune surveillance^53,54^. Pancreatic epithelial cells undergoing malignant transformation produce chemokines (e.g., Ccl2, Ccl4-5) to recruit macrophages and MDSCs to pancreata and thus create an immunosuppressive TME^53,55,56^. Indeed, we found that PPARδ activation by the HFD or GW-upregulated proinflammatory and pro-tumorigenic chemokines (e.g., Ccl2) and increased pancreatic macrophages and MDSC accumulations in pancreata of KC mice, and these events were markedly accelerated by PPARδ hyperactivation in KC/Pd mice. The clinical relevance of these mouse findings was supported by the data from the Oncomine database showing that PPARδ upregulation in human PDACs was associated with upregulation of various pro-tumorigenic chemokines (e.g., CCL2, CCL4-5) and cytokines (e.g., IL6).

Furthermore, our in-depth mechanistic in vivo and in vitro studies and subsequent confirmatory studies in several settings of human and mouse pancreatic epithelial cells revealed PPARδ hyperactivation upregulated Ccl2 expression in pancreatic epithelial cells and increased infiltration of TAMs and MDSCs expressing Ccr2, the primary receptor of Ccl2, into the pancreatic TME to promote progression of pancreatic carcinogenesis in *KRAS*^*mu*^ mice. Recruitment of pancreatic CCR2^+^ TAMs into human pancreata is associated with worse prognosis of human PDAC^43^; targeting TAMs by inhibiting Ccr2 improved chemotherapeutic response in mice^46^. We found that pharmacological inhibition of Ccr2 suppressed recruitment of Ccr2^+^ TAMs and Ccr2^+^ MDSCs into the pancreatic TME and inhibited the progression of pancreatic tumorigenesis by GW-induced PPARδ hyperactivation. Our findings identify PPARδ hyperactivation as a mechanism to trigger CCL2/CCR2-TAMs/MDSCs signaling to subsequently promote the progression of pancreatic tumorigenesis.

In summary, our results demonstrate the strong impact of PPARδ hyperactivation by natural or synthetic ligands in promoting the progression of *KRAS*^*mu*^*-*initiated pancreatic carcinogenesis into PDAC. These findings provide a strong rationale for targeting PPARδ signaling to prevent the progression of PanIN to PDAC.

## Methods

### Antibodies

Information for all the antibodies used in this study are described in Supplementary Table 1.

### Cell lines

One NB490 KPC, two iKPC, and two KPC mouse PDAC cell lines were kindly provided by Drs. Anirban Maitra and Haoqiang Ying at MD Anderson Cancer Center and by David A. Tuveson at Cold Spring Harbor Laboratory, respectively. KC and KC/Pd mouse PDAC cell lines were generated from pancreatic cancer tissues of 50-week-old KC and KC/Pd mice by our laboratory. Briefly, the pancreata were removed from KC or KC/pd male mice at age 50 weeks. After three washings with Hank’s Balanced Salt Solution, the pancreatic tumors were collected, minced into small pieces (~1 mm/strip), and cultured in Dulbecco modified Eagle medium (4.5 g/L glucose) with 10% fetal bovine serum and 1% antibiotic-antimycotic solution (Thermo Fisher Scientific) at 37°C in a humidified incubator in an atmosphere of 5% CO_2_. To remove fibroblast contamination, the attached cells were washed briefly with 0.05% trypsin/EDTA (Thermo Fisher Scientific) for 30 seconds, and then the detached cells were removed. The rest of the attached cells were further digested and transferred into a new flask to culture. From the second passage of primary cultivated cells, the fetal bovine serum concentration in the medium was decreased from 10% to 2% for the cell culture to diminish fibroblast cell contamination. Two mouse pancreatic adenocarcinoma cell lines were successfully established and characterized for further experiments, named as KC and KC/Pd PDAC cell lines. L3.3, L3.9, Colo357, Panc-1, PAU8902, and MDA48 human PDAC cells were kindly provided by Dr. Isaiah Fidler’s laboratory. Mycoplasma was routinely tested for all these cell lines. The mouse and human cell lines were all grown at 37°C in 5% CO_2_ in Dulbecco modified Eagle medium (DMEM) supplemented with 10% fetal bovine serum (FBS) and 1% penicillin–streptomycin unless specified.

### Human pancreas tissue sections and human pancreas tissue microarrays

A set of pancreatic tissue microarrays including paired normal tissue, PanINs, and PDAC were purchased from US Biomax (#BIC14011a).

Human pancreatic tissue samples were collected from two patients who underwent endoscopic ultrasound-guided biopsies of pancreatic neoplastic lesions at MD Anderson Cancer Center and from nine patients who underwent surgical resection of pancreatic neoplastic lesions at University of Michigan (UM). The current study using these tissue samples was approved by The Institutional Review Boards of MD Anderson and University of Michigan. Informed consents were obtained from all participants. De-identified sections from freshly formalin-fixed pancreatic tissues were used for performing hematoxylin and eosin (H&E) staining and PPARδ RNAscope in situ hybridization assays. Histologic assessment for human pancreatic neoplastic lesions was performed by experienced pancreatic pathologists (H.W., J.S.). Two cases from MD Anderson had PanIN 1-2 lesions, and out of 9 cases from University of Michigan, 8 cases had PanIN 1-2 lesions and one case had PanIN 3 lesions.

### Animals

All animal experiments were conducted in accordance with the Guide for the Care and Use of Laboratory Animals and were approved by Institutional Animal Care and Use Committee of MD Anderson with IACUC protocol #00001315. According to the IACUC protocol stipulations, mice were humanely euthanized during studies when they developed one of the following criteria: (1) moribundity, dyspnea, anemia, hunched posture, rough coat, or/and (2) weight loss of more than 20%. *Ppard* conditional overexpression transgenic mice (CAG-LSL-*Ppard*) were generated by pronuclear injection of sequence-confirmed purified DNA fragments of CAG-LSL-*Ppard* at the Genetically Engineered Mouse Facility at MD Anderson, in which a mouse *Ppard* full-length cDNA was subcloned into HindIII/EcoRV sites of CAG-LSL-Kras^G12V^ vector to replace the Kras^G12V^. The resultant vector was digested with PmeI to remove vector sequences, and the DNA fragment of CAG-LSL-*Ppard* driven by a CAG promoter was purified for this pronuclear injection. The same vector backbone has been widely used in generating the transgenic mice except with *Ppard* replacement of *NF-κB*^57^, *IKK2*^58^, or *KLF4*^59^. *Ppard* conditional KO mice, in which *Ppard* exon 4 is flanked with loxP sites (designated as *Ppard*-flox mice), were a gift from Dr. Ronald Evans^32^. B6.FVB-Tg (Pdx1-cre)6Tuv/J (Pdx1-*Cre*, #014647), B6.129S4-Kras^tm4Tyj^/J (LSL-*Kras*^*G12D*^, #008179), and B6.Cg-Gt(ROSA)26Sor^tm14(CAG-tdTomato)Hze^/J (LSL-*tdTomato*, #007914) mice were purchased from the Jackson Laboratory. The genetic background of all the mice used in this study is C57BL/6 J. The mice were housed with a dark/light cycle of 12 h, ambient temperature of 22°C and humidity of 30-70%.

### Generation of experimental mice

1. Pdx1-*Cre* mice were bred with LSL-*Kras*^*G12D*^ or with LSL-*Kras*^G12D^; CAG-LSL-*Ppard* mice to generate Pdx1-*Cre;* LSL-*Kras*^G12D^ mice (called KC mice); or Pdx1-*Cre*; LSL-*Kras*^G12D^; CAG-LSL-*Ppard* mice (called KC/Pd mice), in which *Kras*^*G12D*^ mutation or both *Kras*^G12D^ mutation and *Ppard* overexpression, respectively, were induced specifically in Pdx1-expressing pancreatic epithelial cells.
2. Pdx1-*Cre*; *Ppard*-flox^(+/-)^ mice were bred with LSL-*Kras*^*G12D*^; *Ppard*-flox^(+/-)^ mice to generate Pdx1-*Cre*; LSL-*Kras*^G12D^; *Ppard*-flox^(-/-)^ mice (called KC/PdKO mice), in which *Kras*^*G12D*^ mutation and *Ppard* genetic deletion/KO were induced specifically in Pdx1-expressing pancreatic epithelial cells.
3. LSL-*tdTomato* mice were bred with KC/Pd mice to generate Pdx1-*Cre*; LSL-*Kras*^*G12D*^; LSL-*tdTomato*; CAG-LSL-*Ppard* mice (called KC/tdPd), in which Pdx1-expressing epithelial cells were marked with tdTomato fluorescence protein while *Kras*^*G12D*^ mutation and *Ppard* overexpression were directed into these cells.

### Mouse treatment with HFD or GW501516 diet or CCR2 inhibitor PF4136309 and pancreatic tumorigenesis evaluation

(1) KC or KC/Pd mice at age 6-8 weeks were fed either an HFD (60% kcal from fat; #D12492, Research Diets) or a control diet (10% kcal from fat; #D12450J, Research Diets) for 12 weeks (*n* = 6–10 mice per group). (2) KC or KC/PdKO mice at age 6–8 weeks were fed either the HFD or the control diet for 26 weeks (*n* = 7–9 mice per group). (3) KC or KC/Pd mice at age 6–8 weeks were fed a diet containing 50 mg/kg GW501516 (GW; customized diet, #TD.140302, Envigo) for 0 days (control diet as the same diet except without GW [#TD.110161: 2019 Teklad Global 19% Protein Rodent Diet, Envigo] for 9 days), 3 days (control diet for 6 days and GW diet for 3 days), or 9 days (GW diet for 9 days) (*n* = 5–8 mice per group). The chemical GW501516 was synthesized by the Translational Chemistry Core Facility at MD Anderson Cancer Center, and its authenticity was confirmed by liquid chromatography–mass spectrometry using standard GW (#SML1491, Sigma-Aldrich). (4) KC or KC/PdKO mice at age 6-8 weeks were fed a diet containing 50 mg/kg GW or control diet for 13 weeks (*n* = 5–8 mice per group). (5) KC/Pd mice at age 6–8 weeks were subcutaneously injected with CCR2 inhibitor PF4136309^47^ at a dose of 80 mg/kg or with control solvent (corn oil) twice a day^46^ for 4 days while simultaneously fed the GW diet (50 mg/kg) for the last 3 days (*n* = 5 mice per group). The mice were euthanized after the completion of the treatment, and the pancreas from each mouse was removed, weighed, photographed, and grossly inspected for tumor formation. Half of the pancreas from each mouse was harvested for RNA and protein analyses, and the other half was put in 10% neutral formalin to fix overnight. The formalin-fixed tissues were then embedded in paraffin for further sectioning analysis.

### Mouse survival experiments

The survival experiments were performed on WT, KC or KC/Pd mice at age 6–8 weeks fed a GW (50 mg/kg) or a control diet (*n* = 12-15 mice per group). The mice were followed until they required euthanasia on the basis of one of the following preset criteria with the support of experienced veterinary technologists from our animal facility: (1) moribundity, dyspnea, anemia, hunched posture, rough coat, or/and (2) weight loss of more than 20%.

### Mouse pancreatic tissue histology

Formalin-fixed samples were embedded in paraffin and sectioned onto slides at 5 μm thick and then stained with H&E. Digital HE staining slides for mouse pancreata tissues were scanned with Aperio AT2 (Leica biosystems) and the scanned images were captured with Aperio ImageScope software [v12.3.3.5048]. Histologic assessment for PanIN grade 1–3 and PDAC lesions was performed with the support of experienced pancreatic pathologists (H.W., M.G.,). All foci of PanIN grade 1–3 or PDAC lesion areas were counted, and frequencies of PanIN grade 1–3 and PDAC were then calculated as the percentage of the pancreatic neoplastic area out of the whole pancreas area.

### IHC and immunofluorescence (IF) staining

Tissue sections 5 µm thick were deparaffinized and rehydrated. Antigen retrieval was then performed by immersion of slides into Antigen Unmasking Solution (#H3300, Vector Laboratories) and heating in a steam chamber for 35 min. For IHC staining, slides were treated with 3% H_2_O_2_ solution to reduce endogenous peroxidase, incubated with blocking buffer (5% goat serum in TBST) for 30 min, and then incubated with primary antibodies overnight. The following primary antibodies were used: PPARδ (#ARP38765_T100, 1:100) from Aviva Systems Biology, and phospho-ERK1/2 (#4370S, 1:400), phospho-Stat3 (Tyr705) (#9145S, 1:200), and F4/80 (#70076S,1:200) from Cell Signaling Technology. On the second day, the tissue sections were incubated with biotinylated secondary antibodies (VECTASTAIN ABC kit; Vector Laboratories) for 1 h, followed by incubation with avidin-coupled peroxidase (Vector Laboratories) for 30 min. The slides were developed using 3,3ʹ-diaminobenzidine (DAB) (Agilent Dako) and then counterstained with Mayer’s hematoxylin (Agilent Dako). For IF staining, the slides were incubated with blocking buffer (1.5% goat serum, 0.3% Triton X-100 in phosphate-buffered saline [PBS]) for 1 h at room temperature and then underwent primary incubation at 4°C overnight. The primary antibodies were: α-SMA (#A5228, 1:50, Sigma-Aldrich), cytokeratin 19 (TROMA-III-c, 1:100, Developmental Studies Hybridoma Bank), α-amylase (#A8273, 1:250, Sigma-Aldrich), F4/80 (#71299 S,1:100, Cell Signaling Technology), CCR2 (Ab273050, 1:250, Abcam), and Gr1 (#108402, 1:50, BioLegend). On the second day, the slides were incubated with the Alexa Fluor 488 or Alexa Fluor 594 fluorescence-conjugated secondary antibody at room temperature for 1 h, and then washed and mounted with ProLong Gold Antifade Mountant with DAPI (Thermo Fisher Scientific). For IHC scoring, the intensity of staining was categorized as 1 (weak staining), 2 (moderate staining), or 3 (strong staining), and the final score was calculated by multiplying the percentage of positive cells (0–100) and the intensity (0–3)^60^.

### RNAscope in situ hybridization (RNAscope-ISH) and dual staining of RNAscope-ISH with IHC or with IF

RNA-ISH was performed according to the manufacturer’s manuals (RNAscope 2.5 assay, #322350, Bio-Techne ACDbio). Briefly, fresh paraffin-embedded pancreatic tissue blocks were sectioned into 5 µm per slide the day before the experiment. On the next day, the slides were deparaffinized, then treated with 3% H_2_O_2_ at room temperature for 10 min. The slides were immersed in RNAscope-specific target retrieval reagent (Bio-Techne ACDbio) and boiled in a steam chamber for 15 min. Slides were washed by 100% ethanol at room temperature for 3 min, dried at 60°C for 5 min, and then incubated with protease reagent in the humidity control tray in the HybEZ oven (Bio-Techne ACDbio) at 40°C for 30 min. Next, the hybridization was performed in the HybEZ oven for 2 h using the following probes: mouse Ppard (#504451, Bio-Techne ACDbio), mouse Ccl2 (#311791, Bio-Techne ACDbio), and human PPARδ (#546391, Bio-Techne ACDbio). After the hybridization, the slides were washed, and signal amplification was performed using AMP1-6 reagents (Bio-Techne ACDbio). The final signals were produced by incubation of slides with mixture of Fast RED A and Fast RED B reagents, followed by Mayer’s hematoxylin staining. RNA-ISH intensity was scored as follows: the staining red dots per cell in each slide were counted under 20× or 40× bright-field microscope and then were scored as 0 (no staining or less than 1 dot/10 cells), 1 + (1–3 dots/cell), 2 + (4–10 dots/cell, very few dot clusters), 3 + (>10 dots/cell, and less than 10% positive cells have dot clusters), or 4 + (>10 dots/cell, and more than 10% positive cells have dot clusters) according to the manufacturer’s guideline (SOP 45-006, Bio-Techne ACDbio). Dual staining of RNAscope-ISH with IHC or with IF was performed following the manufacturer’s protocols (Bio-Techne ACDbio). Briefly, RNAscope-ISH was first performed on sections at the step prior to Mayer’s hematoxylin staining. Then, the sections were washed with distilled water and PBST, blocked with 4% goat serum for 1 h at room temperature, and incubated with primary antibodies at 4°C overnight, followed by IHC or IF staining with the same methods as described above.

### Generation of stable PPARδ-overexpressing cell lines

PPARδ-overexpressed cell clones were generated as described before^61^. Briefly, lentivirus plasmids for human *PPARD* ORF (#RC214735L3, OriGene), mouse *PPARD* ORF (#MR207001L3, OriGene) in pLenti-C-myc-DDK-P2A-Puro, and control plasmid pLenti-C-Myc-DDK-P2A-Puro (#PS100092, OriGene) were packaged into lentivirus particles at MD Anderson’s shRNA and ORFeome Core Facility. Human (MDA48 and PAU8902) or mouse (KC, KPC #1, and KPC #2) PDAC cells were transduced with lentiviral particles (10 MOIs for all lentiviruses) with hexadimethrine bromide (8 μg/ml). After 12 h, the culture medium was replaced with fresh medium containing puromycin (4 μg/ml). The medium was changed once every 72 h. Clones with stable transduction were isolated and characterized for DDK-tagged PPARδ expression by Western blot using anti-DDK antibody (#TA50011-100, 1: 2000, OriGene) and then expanded for further analyses.

### Generation of *Ppard* genetic knockout in mouse KPC cells by CRISPR

We first constructed a mouse *Ppard*–targeting CRISPR plasmid by subcloning the following pairs of DNA oligos: 5ʹ-(phos) caccgGAGGAAGTGGCCATGGGTGA-3ʹ (sense) and 5ʹ-(phos) aaacTCACCCATGGCCACTTCCTCc-3ʹ (antisense) into pSpCas9(BB)-2A-Puro (PX459) plasmid (Addgene, #48139) between two BbsI restriction enzyme sites. The mouse *Ppard*–targeting vector or empty vector were transfected into KPC cells and selected by puromycin (3 µg/ml) for 3 days. The transfected cells were split into 96 wells of plates by gradient dilutions to generate single cell–derived individual clones. PPARδ protein expression was measured by Western blot using anti-PPARδ antibody (#Ab8937, 1: 750, Abcam) to characterize the expanded individual clones. One clone with WT *Ppard* and two individual clones with *Ppard* KO were obtained for further analyses.

### RNA extraction and qRT-PCR

The pancreatic tissues from the euthanized mice were injected with RNAlater solution (Thermo Fisher Scientific) at multiple points using a 30-gauge needle syringe. Then, the tissues were cut into tiny pieces (~1 mm), immersed in RNAlater, and then flash-frozen immediately in liquid nitrogen until the tissues were further processed for RNA analysis. For extraction of total RNA, RNAlater-treated pancreatic tissues were quickly homogenized in TRIzol Reagent (#15596018, ThermoFisher Scientific) by mechanical homogenizer on ice, and then total RNA was isolated according to the TRIzol manufacturer’s instructions. A 500-ng aliquot of each RNA sample was reverse transcribed in a 20 μl reaction volume using iScript cDNA synthesis kit (#1708891, Bio-Rad Laboratories). qPCR was carried out in 25 μl of a reaction mixture containing 1 μl of cDNA (25 ng/μl), 12.5 μl of 2× KAPA PROBE FAST Universal 2X qPCR Master Mix (#07959877001, Roche), 10.25 μl double distilled water, and 1.25 μl of 20× TaqMan expression probe. mRNA relative expression levels of Ppard, CK19 (Krt19), amylase (Amy1), Ccl2, Il6, and Tnfa in mouse tissues were measured, and the mouse Ywhaz was used as an endogenous reference. ^62^. Relative quantification of gene expression was analyzed with the Step One Software v2.1 (Applied Biosystem). For mouse and human PDAC cell lines, total RNA extraction and qRT-PCR were performed using the same method as described above for pancreatic tissues. Mouse Actb and human HPRT1 were used as endogenous references for mouse and human cell lines, respectively. TaqMan expression probes’ information for qPCR is described in Supplementary Table 2.

### Protein lysate preparation and Western blot

Soon after mice were euthanized, their pancreatic tissues were flash-frozen by liquid nitrogen in sterile tubes. The frozen tissues were grounded into powder in liquid nitrogen-chilled aluminum foil and mechanically homogenized in protein lysis buffer (0.5% Nonidet P-40, 20 mM 3- [N-morpholino] propanesulfonic acid [pH 7.0], 2 mM ethylene glycol tetraacetic acid, 5 mM ethylenediaminetetraacetic acid, 30 mM sodium fluoride, 40 mM β-glycerophosphate, 2 mM sodium orthovanadate, 1 mM phenylmethylsulfonyl fluoride, and 1× complete protease inhibitor cocktail [Roche Applied Science]). Ponceau S–stained total protein in membrane was used as a loading control. Mouse and human PDAC cell lines were homogenized in the same lysis buffer described above. Forty-microgram samples of each protein were separated onto 7.5–12% sodium dodecyl sulfate polyacrylamide gel. After electrophoresis, the proteins were transferred to a nitrocellulose membrane. The membranes were blocked with 5% milk for 2 h at room temperature and hybridized with anti-mouse PPARD (#ab8937, 1:750, Abcam), anti-phospho-ERK1/2 (#4370 s, 1:2000, Cell Signaling Technology) and anti-phospho-STAT3 (Tyr705) (#9145s, 1:1000, Cell Signaling Technology) at 4 °C overnight. Then, the blots were hybridized with the secondary antibody for 1 h at room temperature. The blots were analyzed using enhanced chemiluminescence (GE Healthcare).

### Active RAS detection by Raf pull-down assay

Two iKPC cell lines cultured with 1 µg/ml doxycycline were treated with 1 µM MEK inhibitor PD0325901 or solvent control (DMSO) for 24 h, and the cells were harvested for measuring KRAS direct activity using an active RAS detection kit (#8821, Cell Signaling Technology) according to the manufacturer’s protocols. Briefly, the cells were rinsed by PBS and scraped in chilled Lysis/Binding/Wash Buffer plus 1 mM PMSF, then incubated on ice for 5 min. After centrifugation at 16,000 *g* at 4 °C for 15 min, supernatant was transferred to a new tube. A total of 100 µl of the 50% glutathione resin slurry was centrifuged in the spin cup with a collection tube at 6,000 *g* for 10-30 s, then resin was further washed by 400 µl of 1X Lysis/Binding/Wash Buffer at 6,000 *g* for 10-30 s, the flow-through was discarded. A total of 80 µg of GST-Raf1-RBD and 500 µg of protein lysate were added to the spin cup containing the glutathione resin, the cap was sealed, and the mixture was incubated at 4°C for 1 h with gentle rocking. After centrifugation and further washing by Lysis/Binding/Wash Buffer at 6,000 *g* at 4 °C for 10-30 s, 50 µl of the reducing sample buffer (2X SDS Sample Buffer, 200 mM DTT) was added to the resin, followed by vortex and centrifugation at 6000 *g* to harvest the eluted samples. Subsequently, the eluted samples were processed for Western blot to measure RAS protein expression levels (#8832 in #8821 [active RAS detection kit], 1:200, Cell Signaling Technology).

### RNA-sequencing for transcriptome profile analyses

The KC and KC/Pd mice at 6–8 weeks were fed a GW diet (50 mg/kg) for 3 days and then euthanized (*n* = 3–4 per group). Total RNA was isolated from the mouse pancreatic tissues using the RNeasy Mini/Micro Kit (Qiagen) according to the manufacturer’s instructions. The RNA quality was measured by the Agilent Bioanalyzer. The total RNA samples with RNA integrity numbers (RINs) ≥7 were processed for RNA-seq transcriptome profile analysis in the Next Generation Sequencing Core at the Science Park, MD Anderson Cancer Center, as described before^41^. Briefly, the TruSeq RNA sample prep kit v2 (Illumina) was used for constructing Illumina-compatible mRNA libraries, and an Illumina HiSeq 3000 instrument was employed for multiplexing and sequencing of the six libraries using the 75-bp paired-end format. After sequencing, the sequencing-generated BCL files were converted into fastq.gz files, and the sample libraries were de-multiplexed using CASAVA 1.8.2 to exclude mismatches. Comparative bioinformatics statistical analyses for the RNA-seq raw data were performed by two experienced experts at the Department of Bioinformatics and Computational Biology at MD Anderson Cancer Center. A gene set enrichment assay for the RNA-seq results was analyzed using the R4.1.0 package “ClusterProfiler” and other associated R packages^24^. The full RNA-seq data were deposited to the NCBI Gene Expression Omnibus public database with the accession # GSE176135.

### Immune cell profiling by flow cytometry

Mouse pancreatic tissues were digested by digestion buffer (1 mg/ml collagenase IV [Sigma-Aldrich] and 10 U/ml DNase I [Sigma-Aldrich] in DMEM) at 37 °C for 30 min. The digested tissues were pushed through a 70-µm cell strainer and washed by 40 ml of DMEM without FBS. The digested cells were resuspended in 37% Percoll, with the same volume of 70% Percoll on the bottom of the tube. After centrifugation at 800 *g* for 20 min with break off, the immune cells were isolated from the 37%/70% interface and rinsed by PBS. The cells were first stained by Zombie UV (BioLegend, #423107, 1:500), then were incubated with a cocktail comprising the following antibodies in cell staining buffer (#420201, BioLegend): anti-CD45 (#103131, 1:150), anti-CD3ε (#100355, 1:60), anti-B220 (#103231, 1:200), anti-Gr1 (#108438, 1:150), anti-CD11b (#101215, 1:250), anti-F4/80 (#123131, 1:400), anti-Ly6C (#128033, 1:40), anti-Ly6G (#127651, 1:80), anti-CD4 (#100405, 1:200), anti-IA/IE (#107607, 1:500, all from BioLegend), and anti-CD8a (#564297, 1:200, BD Biosciences). After washing, the stained cells were submitted for multiple-color flow cytometry analysis. The data were collected on LSR Fortessa X-20 analyzer with FACSDiva software version 8.0 (BD) and analyzed using FlowJo version 10 (BD).

### Cell sorting by flow cytometry with tdTomato RFP

Mouse pancreatic tissues from KC/tdPd mice fed a GW (50 mg/kg) or control diet for 3 days were rinsed by cold PBS, cut into 1-mm pieces, and incubated with digestion buffer (1 mg/ml collagenase IV [Sigma-Aldrich] and 10 U/ml DNase I [Sigma-Aldrich] in DMEM) at 37 °C for 30 min. The digested tissues were passed through a 70-µm cell strainer, washed by DMEM, and resuspended in DMEM containing 1% fetal bovine serum and 2 mM EDTA. Digested cells were then sorted by flow cytometry using td-Tomato RFP and harvested in the fresh complete DMEM medium. The cells were centrifuged at a low speed of 300 *g* and subsequently used for further analyses.

### Quantification of panel of LEGENDplex mouse proinflammatory chemokines and cytokines

The panel profiling of proinflammatory chemokines (BioLegend, #740007) or cytokines (BioLegend, #740150) was performed according to the manufacturer’s protocol. Briefly, the assay uses the principles of a sandwich enzyme-linked immunosorbent assay to quantify soluble analytes using a flow cytometer, which allows simultaneous quantification of 13 mouse chemokines (Ccl2, Ccl5, Cxcl10, Ccl11, Ccl17, Ccl3, Ccl4, Cxcl9, Ccl20, Cxcl5, Cxcl1, Cxcl13, and Ccl22) or 13 cytokines (Il1α, Il1β, Il6, Il10, Il12 p70, Il17a, Il23, Il27, Ccl2, Ifnb, Ifng, Tnfa, and Csf2). The whole-protein lysates of the pancreatic tissue were processed and harvested as described previously for Western blot, except without adding sodium dodecyl sulfate. The data were collected and analyzed on BD FACS Canto II analyzer with FACSDiva software version 8.0 (BD) and analyzed using the LEGENDplex version 8.0 Data Analysis Software (BioLegend). The final results for protein lysates were normalized to their corresponding protein and presented as pg/mg of protein as described before^41^.

### Chromatin immunoprecipitation-quantitative PCR assay (ChIP-qPCR)

Murine KC PDAC cells stably transduced DDK-tagged PPARδ-expressing lentivirus^61^, murine KC PDAC and Panc1 cells were treated with 1 µM GW or solvent for 48 h, and then the cells were harvested for chromatin immunoprecipitation (ChIP) and subsequent q-PCR assays as described before^61^. The murine and human CCL2 gene promoter (−2000 to +500 bp, with transcription start site set as 0) were submitted to the online tool TFBIND (https://tfbind.hgc.jp/), and the calculated PPAR binding sites with matrix scores over 0.73 were considered significant. The following primers for the four predicted *Ppard* binding sites (pPDBS) in the murine Ccl2 promoter were used for ChIP-qPCR:: (1) pPDBS at −642 to −623 bp: 5ʹ-CGTGGAGGTAGCAACGAGAT-3ʹ (sense), 5ʹ-CCTCCTCCTGACTAGTGTCCA-3ʹ (antisense); (2) pPDBS at −1490 to −1471 bp: 5ʹ-GAATTGGGCTAAATCCAGAACA-3ʹ (sense), 5ʹ-TACCTCTTGGGACCCTCCTT-3ʹ (antisense); (3) pPDBS at −1753 to −1734 bp: 5ʹ-CCCTGTCTCGAAAAACCAAA-3ʹ (sense), 5ʹ-TGGTCCTTAGCAGCATTGTG-3ʹ (antisense); and (4) pPDBS at −1838 to −1819 bp: 5ʹ-ACTGCACTGTGAAGGGTTCC-3ʹ (sense), 5ʹ-TCCAGACCTGGAAAGCCATA-3ʹ (antisense). The primers in the human CCL2 promoter were used for ChIP-qPCR: pPDBS at −1534 to −1515 bp: 5’-GGTGGGAGTCTCAGCACATC-3ʹ (sense), 5’-GTGCATTCAGGGAGTCAGGT-3’ (antisense). Input from 1% of total protein from each cell line was used as an endogenous control, and the relative enrichment levels of DDK-tagged PPARδ-binding DNA fragments in Ccl2 promoter using anti-DDK antibody (TA50011-100,1:200, OriGene) or of PPARδ-binding DNA fragments in the mouse or human CCL2 promoter using anti-PPARδ antibody (sc-74517×, 1:500, Santa Cruz Biotechnology) were calculated as 2^−ΔΔCt^. Mouse IgG (#sc-2025, 1:100, Santa Cruz Biotechnology) was used as a negative control. To assess the relative binding activity of PPARδ to the CCL2 promoter regions, relative enriched DDK-tagged PPARδ-binding DNA fragment values or endogenous PPARδ-binding DNA fragment values were calculated by normalization to the readings from control solvent–treated cells.

### Primary pancreatic three-dimensional organoid culture

The mouse pancreatic tissues from KC/tdPd mice treated with the GW diet (50 mg/kg) or control diet for 3 days were digested with digestion buffer (1 mg/ml collagenase IV [Sigma-Aldrich] and 10 U/ml DNase I [Sigma-Aldrich] in DMEM) at 37 °C for 30 min, and the pancreatic epithelial cells were harvested, counted, and embedded in Matrigel (Corning). The Matrigel was topped with organoid culture medium comprising advanced DMEM/F-12 supplemented with penicillin–streptomycin, 2 mM GlutaMAX, 10 mM HEPES (all from Thermo Fisher Scientific), mouse recombinant Wnt-3A at 100 ng/ml (MilliporeSigma), mouse epidermal growth factor at 50 ng/ml (Invitrogen), mouse Noggin at 100 ng/ml (PeproTech), human R-spondin-1 at 1 µg/ml (PeproTech), *N*-acetyl-L-cysteine at 1 mM (Sigma-Aldrich), 1× N-2 supplement (Thermo Fisher Scientific), 1× B-27 supplement (Thermo Fisher Scientific), and Y-27632 at 10 µM (Fisher Scientific). PPARδ agonist GW (1 μM; Sigma-Aldrich) was added to organoid cultures for the mice treated with the GW diet. Organoids were imaged on days 1, 3, and 6 of culture unless otherwise specified. The conditioned culture media were harvested and passed through a 0.45-µm filter to remove the cell debris before being used for further studies.

### MDSCs sorted by flow cytometry from murine immune cells isolated from mouse spleens

To isolate immune cells before sorting, we first harvested spleens from adult C57BL/6 mice and cut the spleens into small pieces (~1 mm). Then we used the plunger base of a syringe to mash the spleens to pass through a 70-µm cell strainer while washing them with RPMI medium (10% FBS) until the spleen was completely mashed. The cells were spun down at 350 *g* for 3 min, and the cell pellets were resuspended in 5 ml of red blood cell lysis buffer (BioLegend) on ice for 5 min, and then 40 ml of cold PBS was added and spun down at 350 *g* for 3 min. The cells were washed with cold PBS once and spun down before being stained with rat PE/Cy7 monoclonal anti-mouse/human CD11b and rat Alexa Fluor 700 anti-mouse Ly-6G/Ly-6C (Gr-1) (BioLegend). CD11b^+^/Gr1^+^ cells (MDSCs) were sorted by flow cytometry and harvested for the chemoattractant assay.

### Chemoattractant assay

The conditioned media collected from organoid culture at day 3 were used for MDSCs’ migration assay. The conditioned media were diluted with equal amounts of fresh RPMI medium before being used for the chemoattractant assay. The migration assay was then performed as follows: a 6.5-mm trans-well with 3-µm pore polycarbonate membrane inserts was added with isolated MDSCs (1×10^5^/well) that were incubated with 600 µl of diluted conditioned media in the wells of a 24-well plate. After 4 h of incubation, MDSCs that passed through the membrane were stained with 0.2% crystal violet in 80% methanol and counted under a ×10 bright-field microscope.

### Quantification and statistical analysis

Statistical significance was determined for one factor in experimental conditions using unpaired Student *t-*test or one-way analysis of variance and Bonferroni adjustments were used for all multiple comparisons. 2-way analysis of variance was used to analyze data involving the simultaneous consideration of 2 factors. Survival time was calculated using the Kaplan–Meier method and compared between groups using the log-rank test. All tests were two-sided, and significance was defined as *P* < .05. Data were analyzed using SAS software v9.4 (SAS Institute) or GraphPad Prism v7.01 (GraphPad Software). The exact *P*-values were provided in Source Data file.

### Reporting summary

Further information on research design is available in the Nature Research Reporting Summary linked to this article.

## Supplementary information

Supplementary Information

Reporting Summary

## Data Availability

A reporting summary for this article is available as Supplementary Information file. The full RNA-seq data generated in this study have been deposited to the NCBI Gene Expression Omnibus public database with the accession # GSE176135. PPARδ mRNA-expression values of iKPC cell lines and iKPC orthotopic tumors were retrieved from the transcriptome-sequencing data in the NCBI Gene Expression Omnibus public database with the accession # GSE32277. Heatmap of human pancreatic chemokines’ and cytokines’ mRNA expression in pancreatic normal and PDAC tissues were retrieved from the transcriptome-sequencing data in the NCBI Gene Expression Omnibus public database with the accession # GSE15471^63^. The data for correlation between PPARδ and CCL2 mRNA expression levels in human PDAC tissues were retrieved from TCGA PanCancer Atlas public database in cBioPortal (https://www.cbioportal.org/). The remaining data are available within the article and its Supplementary Information. Source data are provided with this paper.

## Source data

Source Data
