# Supplementary material for: Rapid acceleration of KRAS-mutant pancreatic carcinogenesis via remodeling of tumor immune microenvironment by PPARδ
Source: Nat Commun. 2022 May 13;13:2665. doi: 10.1038/s41467-022-30392-7 (PMC9106716; doi:10.1038/s41467-022-30392-7)
Supplement: Supplementary file 1 — Supplementary Information [file 41467_2022_30392_MOESM1_ESM.pdf]

Supplementary Figure 1

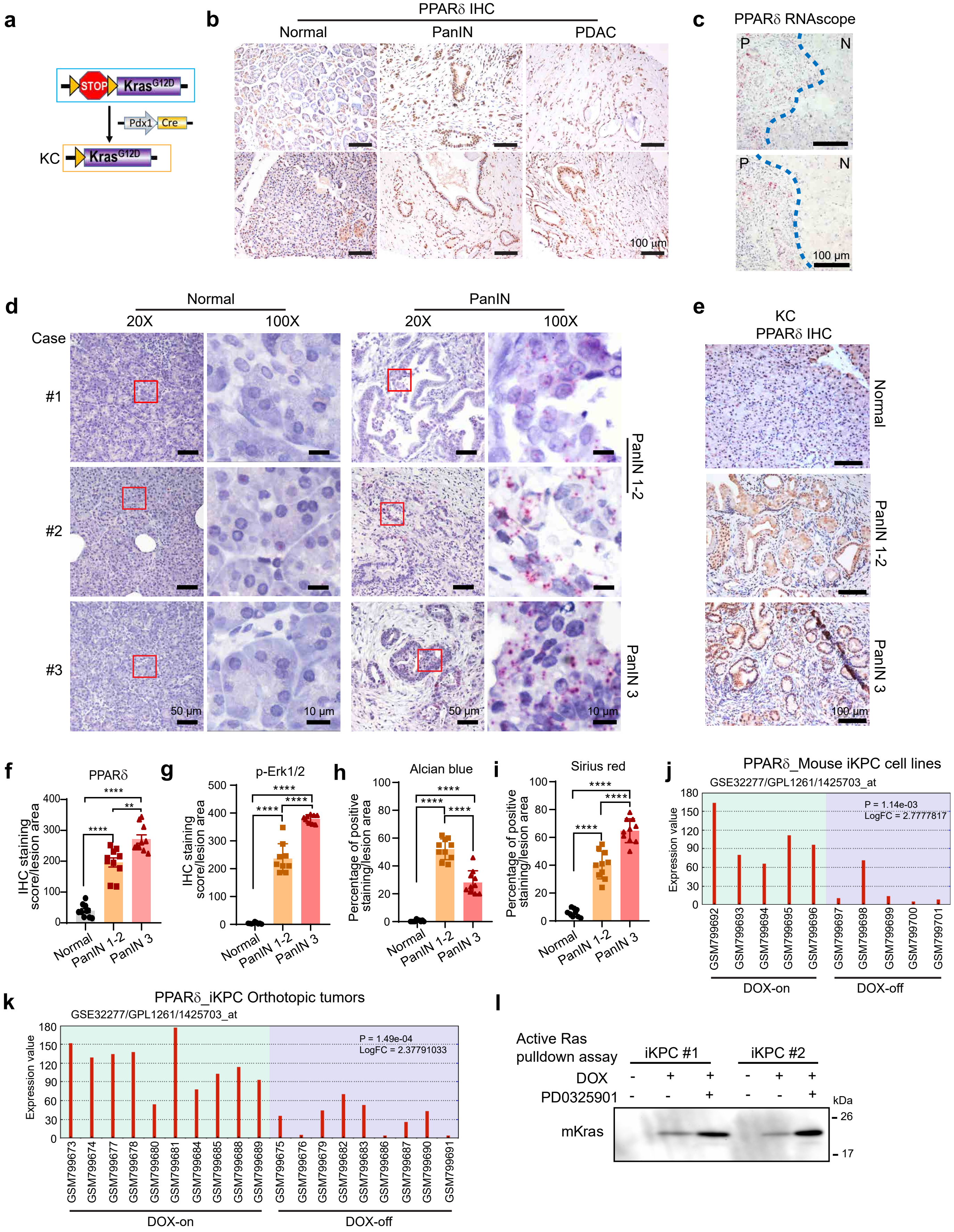

Supplementary Fig. 1. PPARδ expression is upregulated in human and mouse PanINs and regulated by *KRAS*^{mu} activation.

a Schematic diagram for the generation of KC mice. The details are in the Methods subsection "Generation of experimental mice".

b Representative images of PPARδ immunohistochemistry staining for human pancreatic TMA samples with normal tissues (n=11), PanIN 1-2 (n=7), and PDAC (n=6) lesions.

c, d Representative images of PPARδ RNAscope *in situ* hybridizations for sections from human biopsied pancreatic tissue samples, including PanINs (P) and normal (N) areas (n=2) (**c**) and from human surgically resected normal pancreatic areas and PanIN 1-2 (n=8) and PanIN 3 (n=1) (**d**).

e, f Representative images of PPARδ immunohistochemistry staining for mouse pancreatic tissues including normal, PanIN 1-2, and PanIN 3 (**e**) and their quantitative results (**f**) in KC mice (n=10 per group).

g-i Quantitative results of p-Erk1/2 IHC (**g**), Alcian blue (**h**), and Sirius red (**i**) staining for **Fig. 1e** (n=9-10 per group).

j, k Comparisons of PPARδ mRNA expression levels between doxycycline treatment (DOX-on) and control solvent treatment (DOX-off) for five independently cultured iKPC cell lines (**j**) and their orthotopic xenografted tumors (**k**) through GEO2R analyses of mRNA-seq profiling data deposited into the GEO database (#GSE32277).

l Two iKPC mouse PDAC cell lines cultured with 1 μg/ml DOX were treated with 1 μM MEK inhibitor PD0325901 or solvent control (DMSO) for 24 h, and then GTP-bound active Kras was measured by a Raf pulldown assay.

Data are mean ± SEM. For **f-i**, one-way ANOVA with Bonferroni correction. ***P*<.01, and *****P*<.0001. Source data are provided as a Source Data file.

Supplementary Figure 2

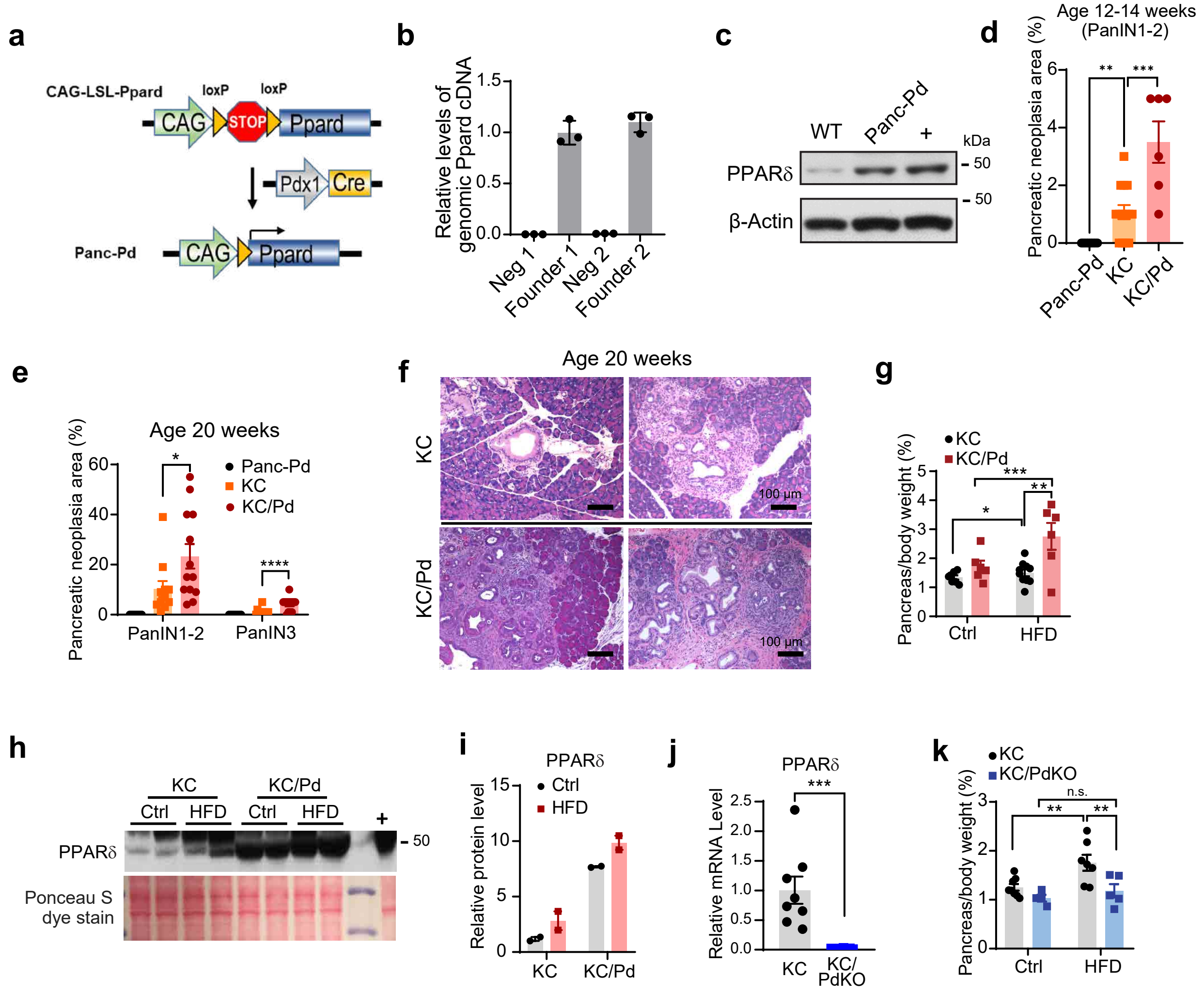

Supplementary Fig. 2. PPARδ hyperactivation by HFD promotes pancreatic tumorigenesis in mice.

a Schematic of the generation of Panc-Pd mice. CAG-LSL-*Ppard* transgenic mice were bred with Pdx1-Cre mice to generate CAG-LSL-*Ppard*; Pdx1-*Cre* mice, designated as Panc-Pd mice.

b, c The relative levels of genomic PPARδ cDNA in tails from 3 independent experiments, measured by qPCR (**b**), and PPARδ protein levels in mouse pancreata, measured by Western blot (**c**), in Panc-Pd mice. Two founders (Founder 1 and 2) for CAG-LSL-*Ppard* transgenic mice were identified. Neg: no detectable *Ppard* cDNA. "+" indicates positive control for PPARδ.

d Comparisons of percentages of PanIN1-2 among Panc-pd (n=10), KC (n=14) and KC/Pd (n=6) mice at age 12-14 weeks.

e, f Histologic characterization of the pancreata in Panc-pd (n=10), KC (n=11) and KC/Pd (n=13) mice at age 20 weeks. (**e**) Comparisons of the percentages of pancreatic neoplasia areas (shown as PanIN 1-2 and PanIN 3) per mouse for the indicated mice. (**f**) Representative images of H&E staining for the indicated mice.

g-i KC and KC/Pd mice at 6-8 weeks old, fed either the HFD or the control diet (Ctrl) for 12 weeks, were euthanized, and pancreata were photographed, weighed, and harvested for gross and histologic characterization. **g** Weight ratio of pancreas to body per mouse (n=6-10 per group). **h** PPARδ protein levels in pancreata, measured by Western blot. Ponceau S dye–stained bands in membrane were evaluated as an internal reference. "+" indicates the PPARδ positive control. **i** Quantitative data for panel **h** (n=2).

j Pancreatic PPARδ mRNA relative expression in pancreata from KC and KC/PdKO mice, measured by qRT-PCR (n=8 per group).

k KC and KC/PdKO mice at 6-8 weeks were fed either the HFD or the Ctrl for 26 weeks and then euthanized. Pancreata were photographed, weighed, harvested, and further analyzed. Weight ratio of pancreas to body per mouse (n= 4-8 per group).

Data are mean ± SEM. For **d**, one-way ANOVA with Bonferroni correction, for **e**, **g** and **k**, two-way ANOVA with Bonferroni correction, and for **j**, unpaired two-tailed Student's *t*-test. *$P<.05$, **$P<.01$, and ***$P<.001$, ****$P<.0001$; n.s.: no significance. Source data are provided as a Source Data file.

Supplementary Figure 3

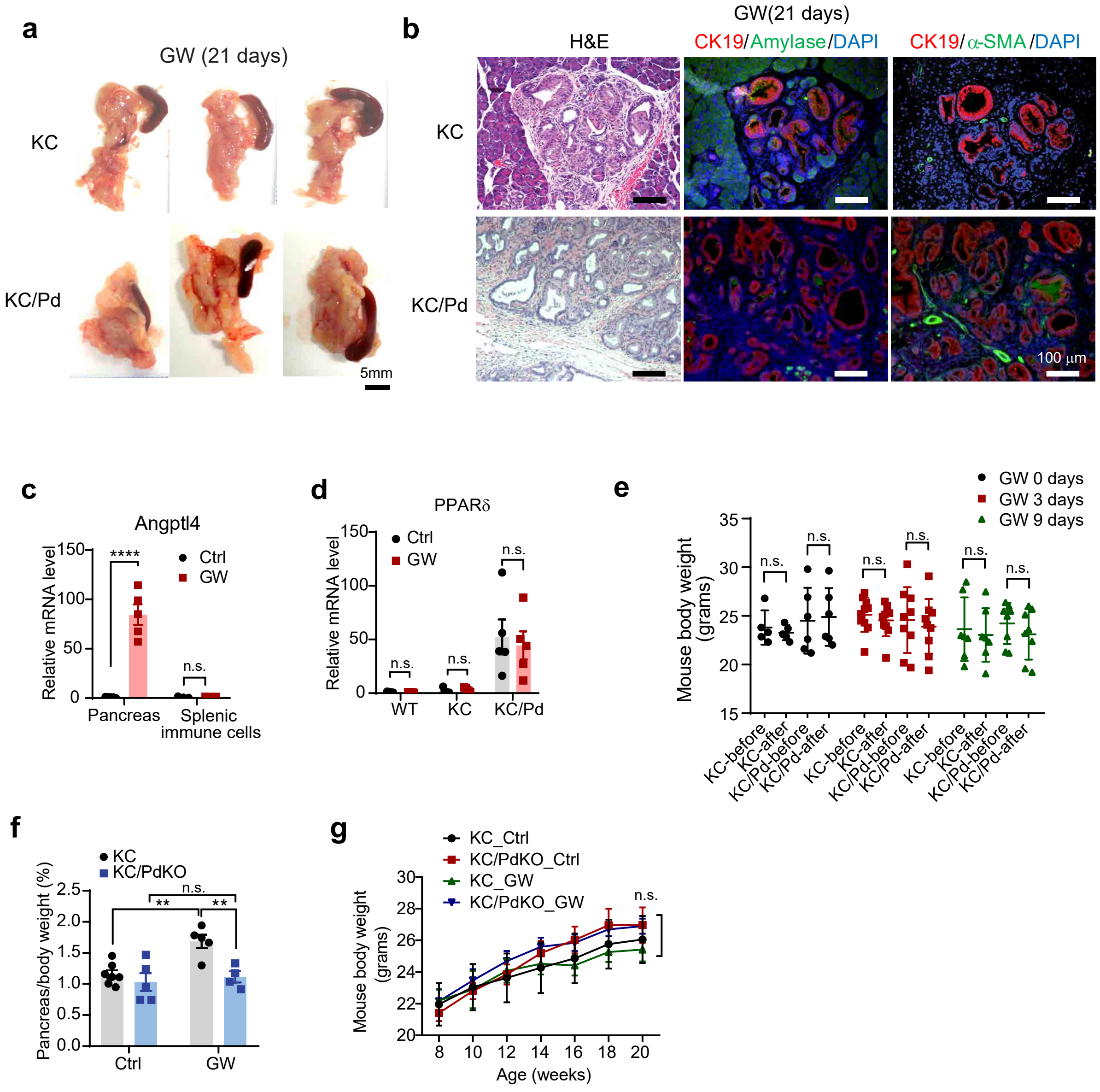

Supplementary Fig. 3. PPARδ hyperactivation by GW501516 (GW) diet promotes pancreatic tumorigenesis in mice.

a, b Representative images (**a**) and H&E staining and co-immunofluorescence staining of CK19 and α-SMA or CK19 and amylase (**b**) of pancreata of KC and KC/Pd mice fed the GW diet for 21 days.

c Angptl4 expression levels of pancreas and isolated splenic immune cells from KC/Pd mice fed the GW or the Ctrl diet for 3 days (n= 3-5 biologically independent samples).

d PPARδ mRNA expression levels for WT, KC, and KC/Pd mice on the GW or control diet for 9 days (n=5 biologically independent samples).

e Comparisons of body weights for KC and KC/Pd mice before and after treatments with the GW diet for 0 days (control diet for 9 days), 3 days (control diet for 6 days and GW diet for 9 days), and 9 days (n=5-10 per group).

f, g. KC and KC/PdKO mice at 6-8 weeks were fed either the GW (50 mg/kg) or the Ctrl diet, weighed once every other week, and then euthanized after 13 weeks of the diet treatment. Pancreata were photographed, weighed, harvested, and further analyzed. **f** Weight ratio of pancreas to body per mouse (n=4-7 per group).

g Body weight curves for KC and KC/PdKO mice on GW or control diet (n= 5-8 per group).

Data are mean ± SEM. For **c-e**, multiple *t*-test, and for **f**, two-way ANOVA with Bonferroni correction, and for **g**, one-way ANOVA with Bonferroni correction. $^{**}P<.01$, and $^{****}P<.0001$; n.s.: no significance.

Source data are provided as a Source Data file.

Supplementary Figure 4

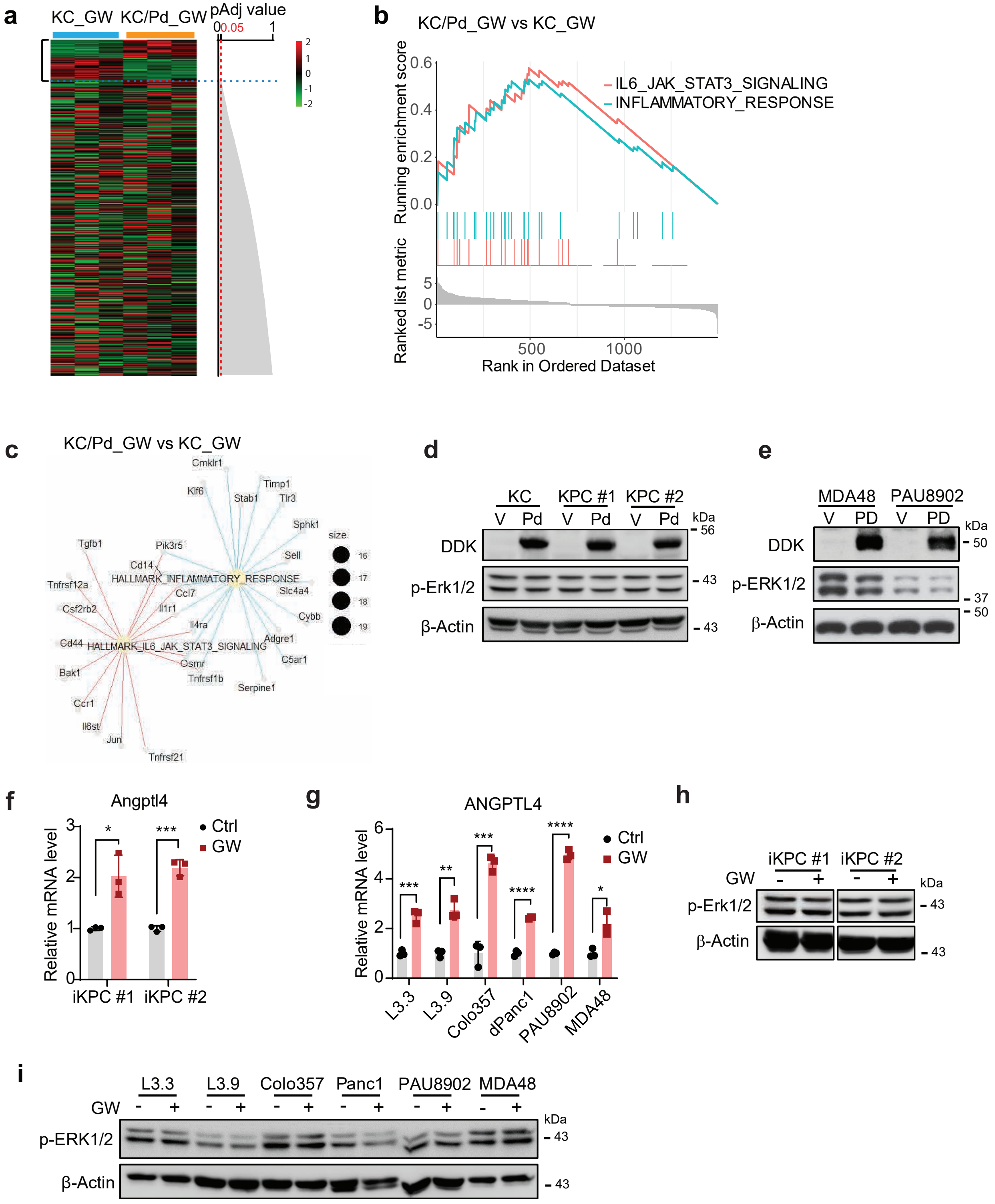

Supplementary Fig. 4. PPARδ hyperactivation by GW activates inflammatory and ERK pathways in the pancreas.

a Heatmap of all expressed genes identified from pancreatic tissues of the GW diet–treated KC (KC_GW) and KC/Pd (KC/Pd_GW) mouse groups by RNA-seq profiling assay as described in Fig. 4a. Genes with *P*(Adj)<0.05 were sorted on the top, and all detectable genes are shown in the order of *P*(Adj) value from low to high (range from 0 to 1).

b, c Gene set enrichment analysis (GSEA) results of KC/Pd_GW versus KC_GW using R package ClusterProfiler, *P*(Adj) cutoff=0.05, gene sets=MSigDB category “Hallmark gene sets”. **b** GSEA plots of the two enriched inflammation-related pathways. **c** The Cnet plots of the same two inflammation-related pathways.

d, e Effects of PPARδ overexpression on $KRAS^{mu}$ activity levels (p-ERK1/2) in mouse and human PDAC cells. Three independent mouse PDAC cell lines (one KC and two KPC cell lines) and two human PDAC cell lines (MDA48 and PAU8902) were stably transduced with mouse DDK-tagged PPARδ (Pd) or human DDK-tagged PPARδ (PD) lentivirus particles, respectively. The cells stably transduced with control lentivirus particles (V) were used as controls. DDK-tagged PPARδ and p-ERK1/2 protein expression levels for mouse (**d**) and human (**e**) PDAC cells were measured by Western blot. Anti-DDK antibody was used for measuring DDK-tagged PPARδ expression levels.

f-i Effects of GW treatment on ANGPTL4 expression and $KRAS^{mu}$ activity levels (measured by p-ERK1/2) in mouse and human PDAC cells. The two indicated mouse iKPC cell lines under DOX treatment (1 µg/ml) and six indicated human PDAC cell lines were treated with 1 µM GW or equal volume of solvent (DMSO) for 48 h. ANGPTL4 mRNA relative expression and p-ERK1/2 protein expression from 3 independent experiments for mouse (**f, h**) and human (**g, i**) PDAC cells were measured by qRT-PCR and Western blot, respectively.

Data are mean ± SEM. For **f** and **g**, multiple *t*-test. *P<.05, **P<.01, ***P<.001, and ****P<.0001. Source data are provided as a Source Data file.

Supplementary Figure 5

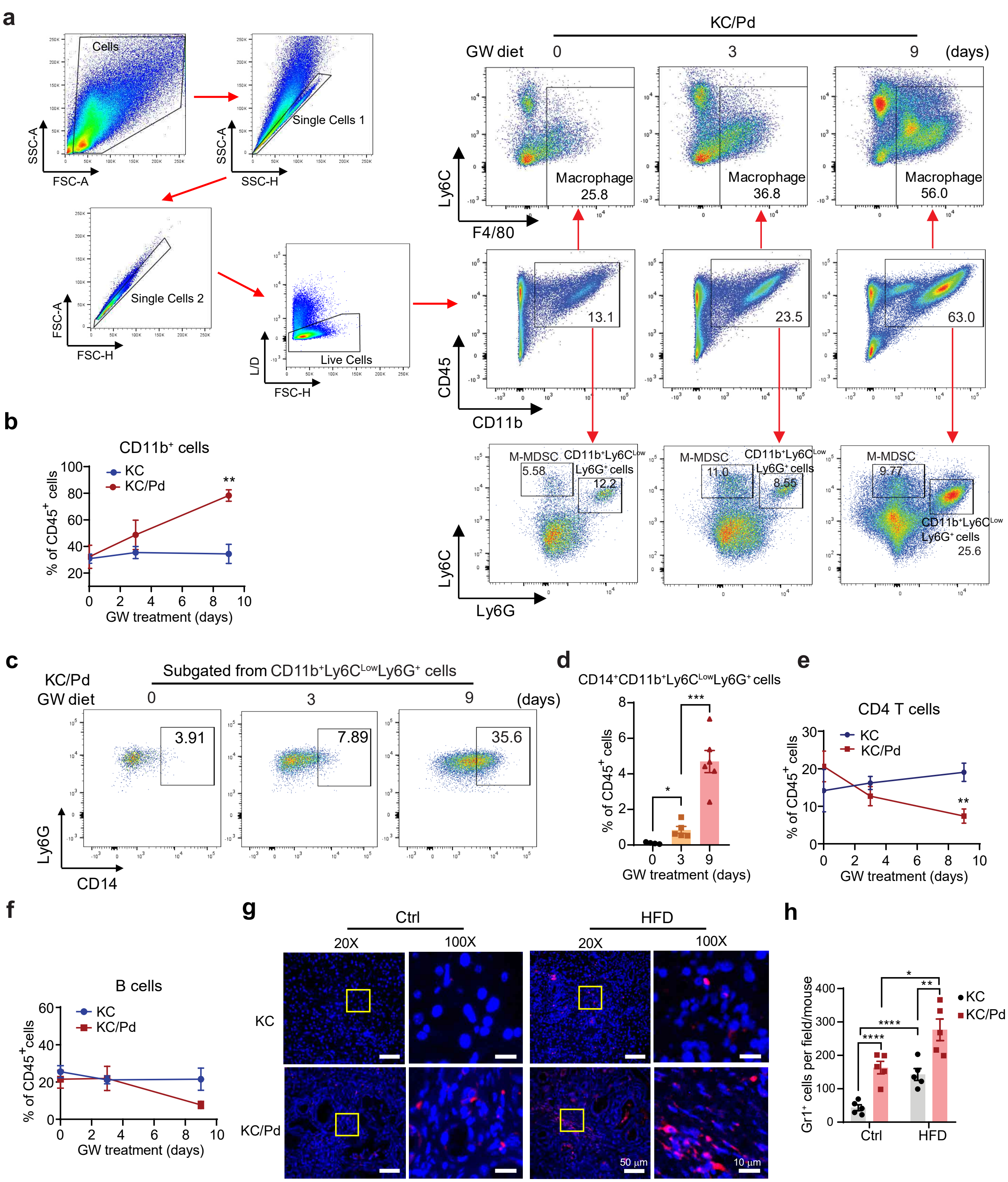

Supplementary Fig. 5. PPARδ hyperactivation by GW or HFD recruits macrophages and myeloid-derived suppressor cells (MDSCs) into the pancreata to remodel TME.

a-f KC and KC/Pd mice at 6-8 weeks were fed the GW diet (50 mg/kg) for 0, 3, or 9 days and then euthanized. Their pancreata were examined for infiltrating immune cells assessed by flow cytometry as described in **Fig. 5a-h** (n=4-6 per group). **a** The representative images of flow cytometry gating strategy and multiple panels for $CD45^{+}CD11b^{+}$ (right middle), macrophages ($CD11b^{+}F4/80^{+}$, right top), M-MDSCs ($CD11b^{+}Ly6C^{hi}Ly6G^{-}$) and $CD11b^{+}Ly6C^{low}Ly6G^{+}$ cells (right bottom). **b-f** Quantitative results of $CD11b^{+}$ cells (**b**), the representative images (**c**) and their quantitative results (**d**) of PMN-MDSCs ($CD14^{+}CD11b^{+}Ly6C^{low}Ly6G^{+}$), quantitative results of $CD3^{+}CD4^{+}$ T cells (**e**), and B cells ($B220^{+}$) (**f**). All quantitative data are presented as percentage of pancreas-infiltrating $CD45^{+}$ cells for the indicated mouse groups (n=4-6 per group).

g, h Representative images of IF staining for MDSCs ($Gr1^{+}$) (**g**) and their quantitative results (**h**) in pancreata for KC and KC/Pd mice fed the HFD or Ctrl for 12 weeks, as described in **Fig. 2b** (n=5 per group).

Data are mean ± SEM. For **b, e** and **f**, multiple *t*-test, for **d**, one-way ANOVA with Bonferroni correction, and for **h**, two-way ANOVA with Bonferroni correction. *P<.05, **P<.01, ***P<.001 and ****P<.0001.

Source data are provided as a Source Data file.

Supplementary Figure 6

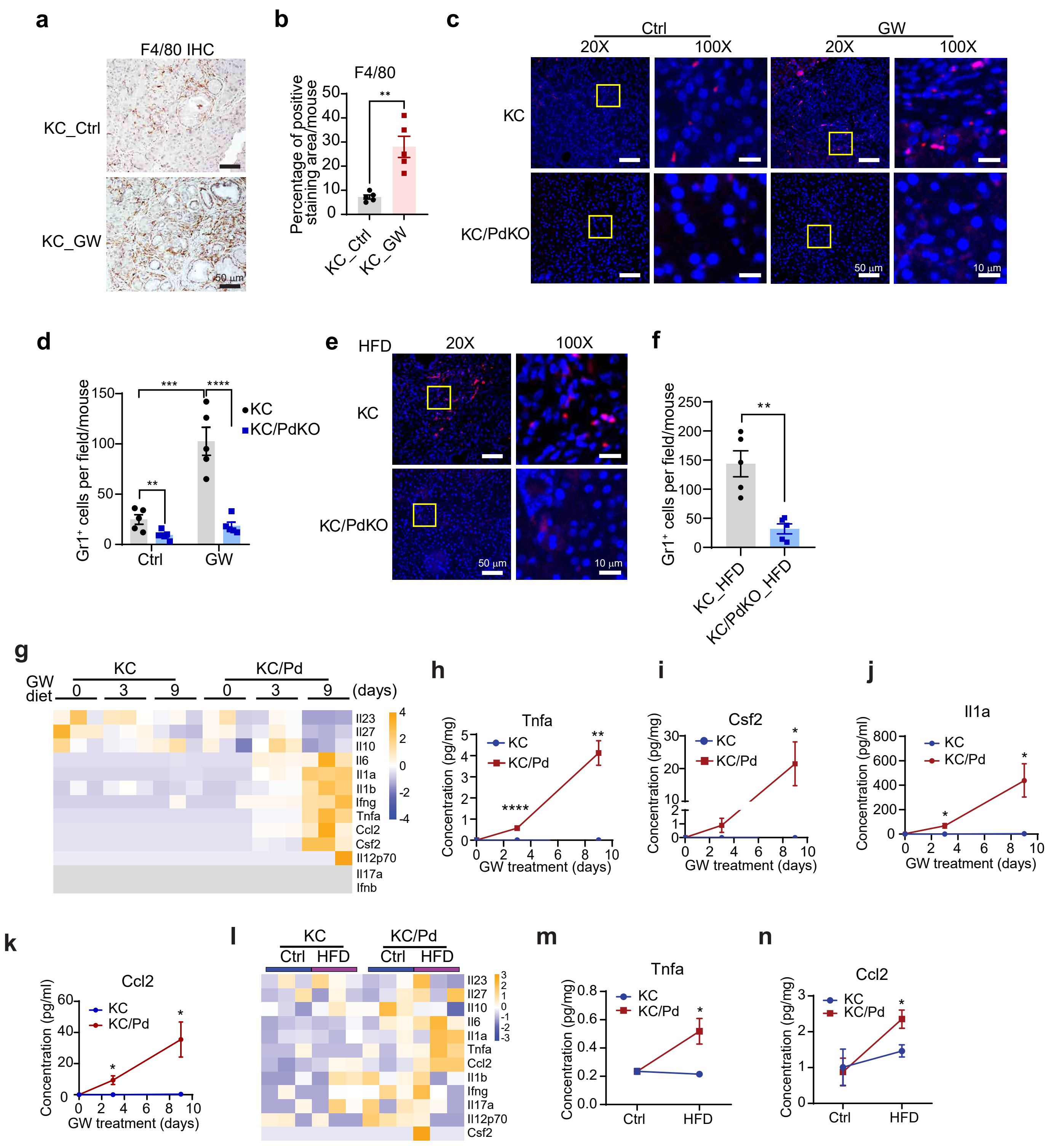

Supplementary Fig. 6. *Ppard* KO suppresses GW- or HFD-increased accumulation of macrophages (F4/80$^+$) and MDSCs (Gr1$^+$), while PPARδ hyperactivation by GW or HFD increases inflammatory cytokines in KC/Pd mice.

a, b Representative IHC staining images for macrophages (F4/80$^+$) (**a**) and their quantitative results (**b**) in pancreata for KC mice fed GW or control for 13 weeks, as described in **Fig. 3h** (n=5 per group).

c, d Representative IF staining images for MDSCs (Gr1$^+$) (**c**) and their quantitative results (**d**) in pancreata for KC and KC/PdKO mice fed GW or control for 13 weeks, as described in **Fig. 3h** (n=5 per group).

e, f Representative IF staining images of MDSCs (Gr1$^+$) (**e**) and their quantitative results (**f**) in pancreata for KC and KC/PdKO mice fed the HFD for 26 weeks, as described in **Fig. 2i** (n=5 per group).

g-n KC and KC/Pd mice at 6-8 weeks were fed the GW diet (50 mg/kg) for 0, 3, or 9 days (**g-k**), or the HFD or control (Ctrl) diet for 12 weeks (**l-n**), and then euthanized. The pancreas tissue protein lysates were examined for a panel of 13 cytokines by BioLegend's LEGENDplex bead-based immunoassays (n=3-5 per group). Heatmap (**g**) and protein concentrations from the indicated mice fed the GW or control diet for Tnfa (**h**), Csf2 (**i**), Il1a (**j**), and Ccl2 (**k**) are shown (n=3-4 per group). Heatmap (**l**) and protein concentrations of the indicated mice fed the HFD or Ctrl diet for Tnfa (**m**) and Ccl2 (**n**) are shown (n=3-4 per group).

Data are mean ± SEM. For **b, f, m** and **n**, unpaired two-tailed Student's *t*-test, for **h-k**, multiple *t*-test, and for **d**, two-way ANOVA with Bonferroni correction. *P<.05, **P<.01, ***P<.001, and ****P<.0001. Source data are provided as a Source Data file.

Supplementary Figure 7

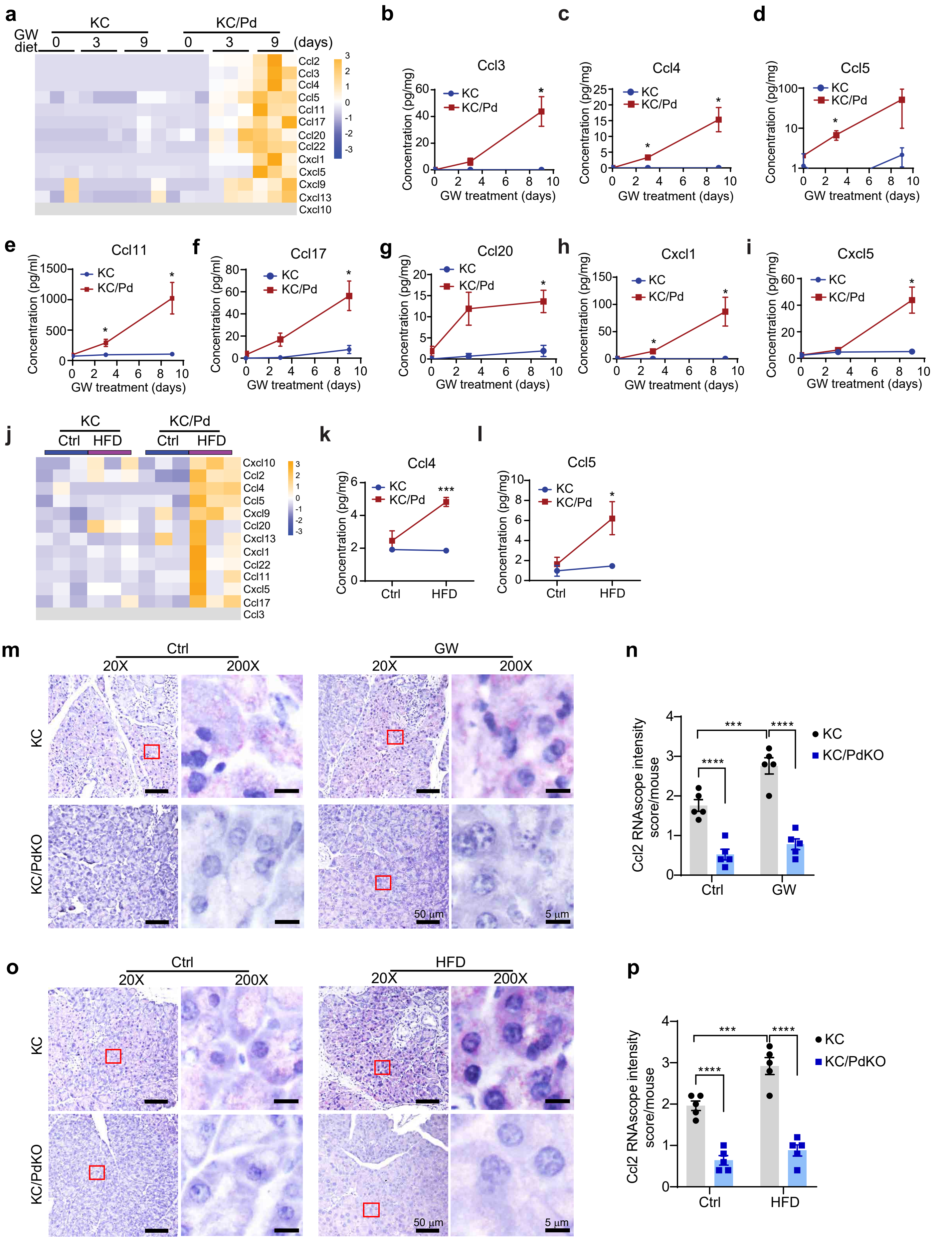

Supplementary Fig. 7. PPARδ hyperactivation upregulates chemokine CCL2 expression in mouse pancreatic tissues.

a-i KC and KC/Pd mice at 6-8 weeks were fed GW (50 mg/kg) for 0, 3, or 9 days and then euthanized. Pancreata were harvested and processed for measurements of a panel of 13 chemokines using BioLegend's LEGENDplex bead-based immunoassays. **a** Heatmaps of 13 screened chemokines from 3 representative mice per group are shown. **b-i** Quantitative results for chemokines, including Ccl3 (**b**), Ccl4 (**c**), Ccl5 (**d**), Ccl11 (**e**), Ccl17 (**f**), Ccl20 (**g**), Cxcl1 (**h**), and Cxcl5 (**i**) are shown (n=3-4 per group).

j-l KC and KC/Pd mice at 6-8 weeks fed either the HFD or the Ctrl diet for 12 weeks were euthanized. Pancreata were harvested and processed for measurement of the same panel of chemokines as described in panels **a-i** (n=3-4 per group). **j** Heatmap of 13 screened chemokines is shown. **k, l** Quantitative results for chemokines including Ccl4 (**k**) and Ccl5 (**l**) are shown.

m, n Representative Ccl2 RNAscope *in situ* hybridizations (**m**) and their quantitative results (**n**) in normal areas of pancreata of KC and KC/PdKO mice fed the GW or Ctrl diet for 13 weeks, as described in **Fig. 3h** (n=5 per group).

o, p Representative Ccl2 RNAscope *in situ* hybridization (**o**) and their quantitative results (**p**) in normal areas of pancreata for KC and KC/PdKO mice fed the HFD or Ctrl for 26 weeks, as described in **Fig. 2i** (n=5 per group).

Data are mean ± SEM. For **b-i,** multiple *t*-test**,** for **k** and **l,** unpaired Student's *t*-test, and for **n, p,** two-way ANOVA with Bonferroni correction. *P<.05, ***P<.001, and ****P<.0001. Source data are provided as a Source Data file.

Supplementary Figure 8

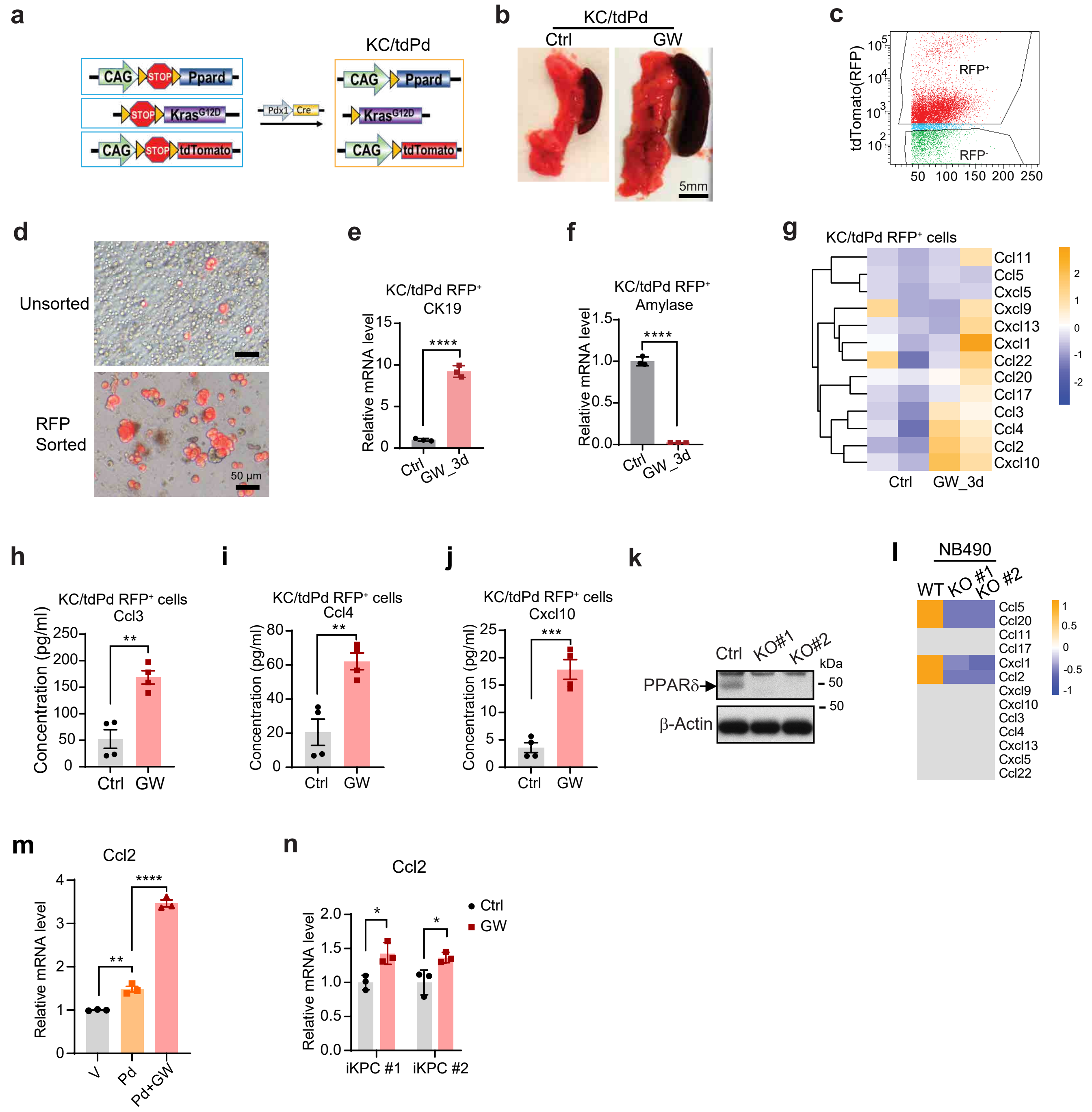

Supplementary Fig. 8. PPARδ hyperactivation by GW upregulates chemokine Ccl2 expression in mouse pancreatic epithelial cells.

a Schematic diagram of the generation of KC/tdPd mice.

b Representative photos of pancreata of KC/tdPd mice fed the GW or Ctrl diet for 3 days.

c Representative images of pancreatic digested cells sorted by flow cytometry with tdTomato red fluorescent protein (RFP).

d Merged images with bright-field light and tdTomato-RFP of the digested pancreatic cells before and after sorting.

e, f Characterization of sorted tdTomato-RFP–marked pancreatic epithelial cells. Pancreatic ductal epithelial marker CK19 (**f**) and acinar cell marker amylase (**g**) mRNA expression levels were measured by qRT-PCR (n=3 per group).

g-j The sorted tdTomato-RFP^{+} pancreatic epithelial cells were processed for measurements of a panel of 13 chemokines as described in **Supplementary Fig. 7a-i** (n=3-4 per group). **g** Heatmaps of 13 screened chemokines from two representative mice per group are shown. **h-j** Quantitative results for chemokines including Ccl3 (**h**), Ccl4 (**i**), and Cxcl10 (**j**) are shown.

k PPARδ protein expression levels in mouse NB490 KPC cells with *Ppard* WT (WT) and with *Ppard*-KO by CRISPR (KO#1 and KO#2 for two individual clones). (n= 3 repeated experiments with similar results).

l Heatmaps of 13 screened chemokines in cell culture media from mouse NB490 KPC cells with *Ppard* WT (WT) and with *Ppard*-KO**.**

m Mouse KC PDAC cell lines stably transduced with control (V) or mouse DDK-tagged PPARδ lentivirus particles (Pd) were treated with 1 µM GW or equal amount of solvent for 48 h and then harvested. Ccl2 mRNA expression levels from 3 independent samples were measured by qRT-PCR.

n Two mouse iKPC cell lines under DOX challenge (1 µg/ml) were treated with 1 µM GW or equal volume of solvent DMSO (Ctrl) for 48 h and then harvested. Ccl2 mRNA expression levels from 3 independent samples were measured by qRT-PCR.

Data are mean ± SEM. For **e**, **f** and **h-j**, two-tailed Student's *t*-test, for **m**, one-way ANOVA with Bonferroni correction, and for **n**, multiple *t*-test. *P<.05, **P<.01, ***P<.001, and ****P<.0001. Source data are provided as a Source Data file.

Supplementary Figure 9

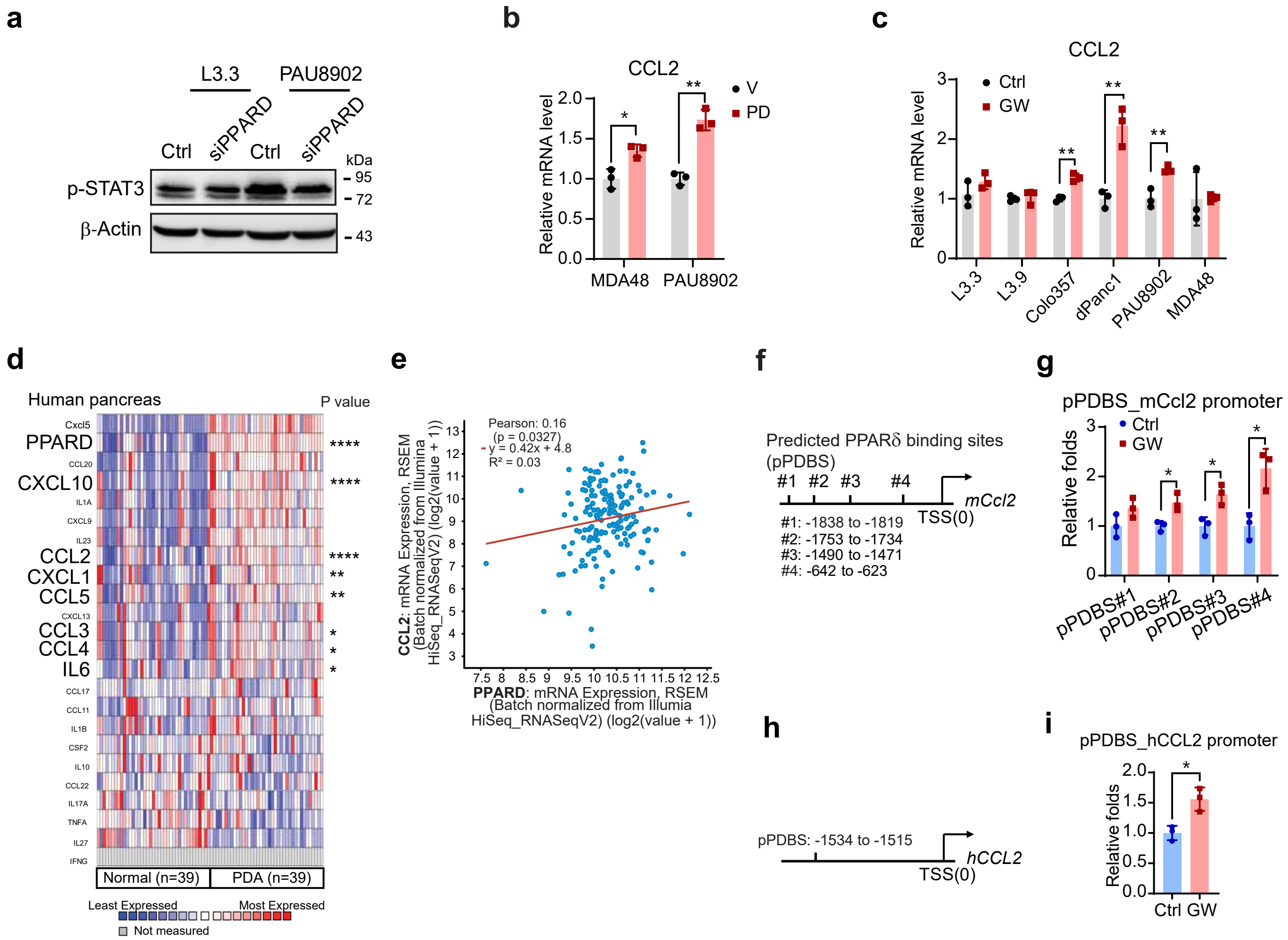

Supplementary Fig. 9. PPARδ activation increases CCL2 expression in human PDAC cells and enhances PPARδ binding to the CCL2 promoter in mouse and human PDAC cells.

a p-STAT3 expression levels in the two indicated human PDAC cell lines transfected with a pool of two PPARδ siRNAs (siPPARD) or control siRNA (Ctrl) for 48 h, measured by Western blot.

b, c CCL2 mRNA expression levels in the two indicated human PDAC cell lines (MDA48 and PAU8902) stably transduced with control (V) or human DDK-tagged PPARδ (PD) (**b**) from 3 independent samples and in the six indicated human PDAC cell lines treated with 1 µM GW or equal volume of its control solvent DMSO (Ctrl) for 48 h (**c**) from 3 independent samples, measured by qRT-PCR.

d Heatmap of human pancreatic chemokines' and cytokines' mRNA expression in pancreatic normal and PDAC tissues from the Oncomine Badea database. Enlarged symbols show genes (i.e., PPARD, CCL2, CCL3-5, CXCL1, CXCL10, IL6) that were in agreement with mouse findings.

e Positive correlation between PPARδ and CCL2 mRNA expression levels in human PDAC tissues from Pan-Cancer Atlas public database analyses using TCGA data (n=168).

f Schematic diagram of the four predicted PPARδ binding sites (pPDBS) in the promoter region of mouse Ccl2 (mCcl2) according to TFBIND online software. TSS, transcription start site.

g The PPARδ binding to the predicted PPARδ binding sites (pPDBS) in the mCcl2 promoter from 3 independent samples, measured by a chromatin immunoprecipitation–quantitative PCR assay in mouse KC PDAC cells treated with 1 µM GW or solvent (DMSO) for 48 h.

h Schematic diagram of the predicted PPARδ binding site in the promoter region of human CCL2 (hCCL2) according to TFBIND online software. TSS, transcription start site.

i The PPARδ binding to the predicted PPARδ binding site (pPDBS) in the hCCL2 promoter shown in panel **p,** from 3 independent samples, measured by a chromatin immunoprecipitation–quantitative PCR assay in human Panc1 PDAC cells treated with 1 µM GW or solvent (DMSO) for 48 h.

Data are mean ± SEM. For **b**, **c**, **d** and **g**, multiple *t*-test, and for **i**, unpaired two-tailed Student's t-test. **P*<.05, and ***P*<.01. Source data are provided as a Source Data file.

Supplementary Figure 10

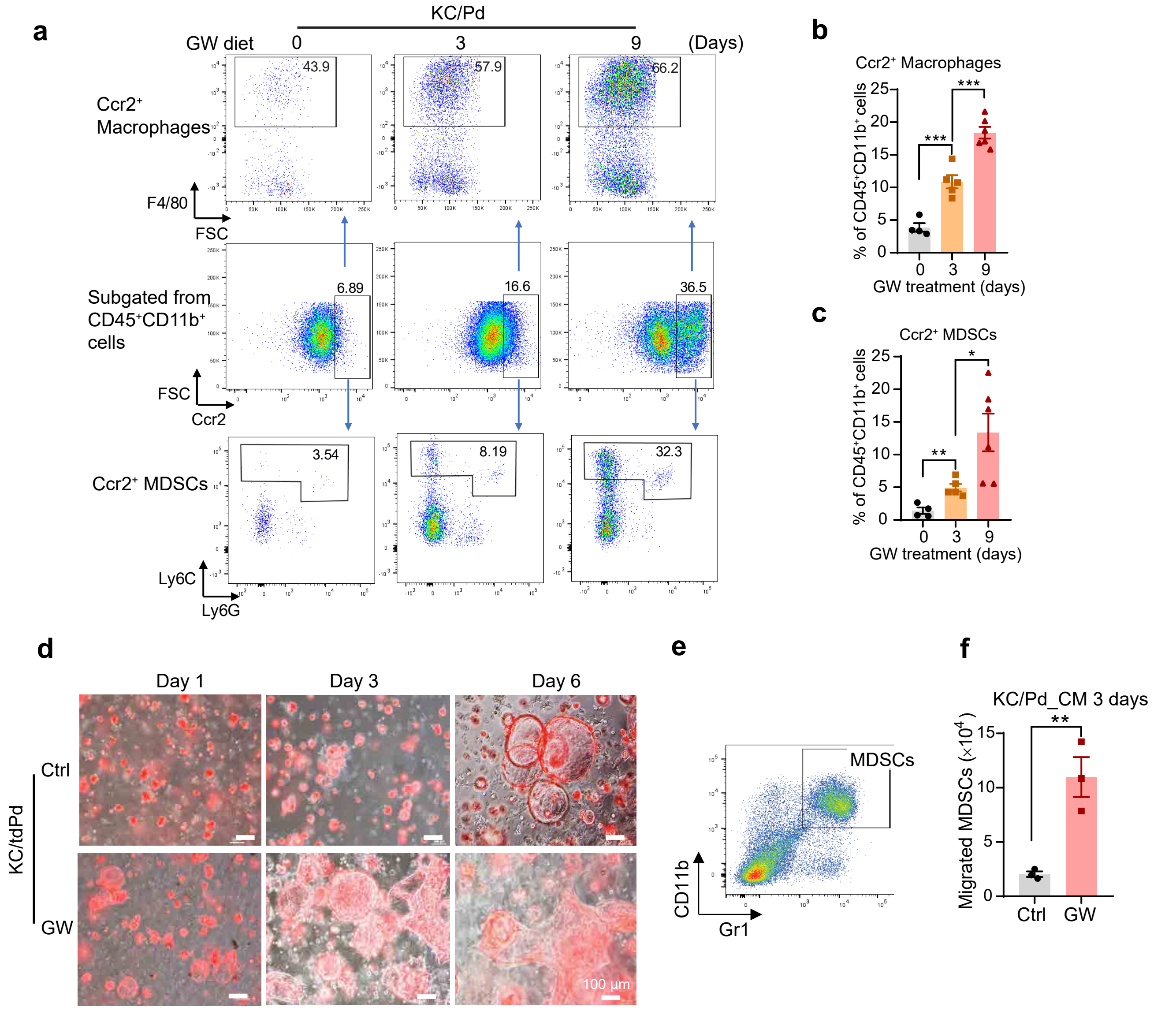

Supplementary Fig. 10. PPARδ hyperactivation–upregulated Ccl2 in *KRAS^{mu}* pancreatic epithelial cells increases pancreatic infiltration of Ccr2^{+} TAMs or Ccr2^{+} MDSCs *in vivo* and increases MDSCs' migration *in vitro*.

a-c KC and KC/Pd mice at 6-8 weeks on the GW diet (50 mg/kg) for 0, 3, or 9 days were euthanized, and then pancreata were examined for quantifications of infiltrating immune cells by flow cytometry (n=4-6 per group). **a** Representative images of multiple panels of flow cytometry for Ccr2^{+} CD11b^{+} immune cells (middle), Ccr2^{+} TAMs (Ccr2^{+}F4/80^{+}, top), and Ccr2^{+} MDSCs (Ccr2^{+}Ly6C^{+} plus Ccr2^{+}Ly6G^{+}, bottom). **b, c** Percentages of Ccr2^{+} TAMs (**b**) and Ccr2^{+} MDSCs (**c**) among pancreas-infiltrating CD45^{+}11b^{+} cells for the indicated mouse groups.

d-f KC/tdPd mice at 6-8 weeks were fed the GW (50 mg/kg) or control (Ctrl) diet for 3 days and then euthanized. The pancreata were enzymatically digested and sorted as described in **Supplementary Fig. 8b-d**, and the sorted tdTomato-RFP^{+} pancreatic epithelial cells were harvested for 3-dimensional (3-D) primary organoid culture. GW (1 µM) was added into culture media for the mice fed the GW diet. The images of 3-D primary cultured organoids were photographed at 1, 3, and 6 days of culture. **d** Representative merged images with bright-field light and tdTomato-RFP of 3-D primary pancreatic organoids at the indicated time points of the culture. **e, f** MDSCs' migration towards conditioned medium (CM) harvested from primary pancreatic organoid culture. The CM were harvested at 3 days of the organoid culture. **e** MDSCs (CD11b^{+}Gr1^{+}) were isolated from spleens of C57BL/6 WT mice by flow cytometry. Representative flow cytometry images of sorted MDSCs are shown. **f** Quantitative results for migrated MDSCs for the indicated mouse groups are shown (n=3 per group).

Data are mean ± SEM; For **b** and **c**, one-way ANOVA with Bonferroni correction, and for **f,** unpaired two-tailed Student's *t*-test. *P<.05, **P<.01, and ***P<.001. Source data are provided as a Source Data file.

Supplementary Table 1. The antibodies used in this study are summarized

Name	Source	Catalog number	Application/Dilution
Rb anti–α-amylase pAb	Sigma-Aldrich	A8273	IF (mm)/1:250
Rat anti-CK19 mAb	Developmental Studies Hybridoma Bank	TROMA-III-c	IF (mm)/1:100
Mm anti–α-SMA mAb	Sigma-Aldrich	A5228	IF (mm)/1:50
Mm anti-DDK mAb	OriGene	TA50011-100	ChIP (mm)/1:200<br>WB (hs/mm)/1:2000
Mm anti-PPARδ mAb	Santa Cruz Biotechnology	sc-74517	ChIP (hs/mm)/1:500
Normal mouse IgG	Santa Cruz Biotechnology	sc-2025	ChIP (hs/mm)/1:100
Rb anti–phospho-ERK1/2 mAb	Cell Signaling Technology	4370S	IHC (hs/mm /1:400<br>WB (hs/mm)/1:2000
Rb anti-phospho-Stat3 (Tyr705) mAb	Cell Signaling Technology	9145S	IHC (hs/mm)/1:200<br>WB (hs/mm)/1:1000
Rb anti-PPARδ pAb	Abcam	Ab8937	WB (mm)/1:750
Mm anti-Ras mAb in active Ras detection kit	Cell Signaling Technology	8821	WB (mm)/1:200
Mm anti–β-Actin mAb	Santa Cruz Biotechnology	sc-47778	WB (hs/mm)/1:1000
Rb anti-PPARδ pAb	Aviva Systems Biology	ARP38765_T100	IHC (hs/mm)/1:100
Rat anti-F4/80 mAb	Cell Signaling Technology	71299S	IF (mm)/1:100
Rb anti-F4/80 mAb	Cell Signaling Technology	70076S	IHC (mm)/1:200<br>Dual ISH-IHC (mm)/1:200
Rb anti-CCR2 mAb	Abcam	Ab273050	IF (mm)/1:250<br>Dual ISH-IF (mm)/1:250
Rat anti-Gr1 mAb	BioLegend	108402	IF (mm)/1:50
Rat PE anti-IA/IE mAb	BioLegend	107607	F (mm)/1:500
Rat PerCP/Cy5.5 anti-CD45 mAb	BioLegend	103131	F (mm)/1:150
Rat Brilliant Violet 785 anti-CD3ε mAb	BioLegend	100355	F (mm)/1:60
Rat Alexa Fluor 700 anti-CD45R/B220 mAb	BioLegend	103231	F (mm)/1:200
Rat PE/Cy7 anti-CD11b mAb	BioLegend	101215	F (mm)/1:250
Rat Alexa Fluor 700 anti-Ly-6G/Ly-6C (Gr-1) mAb	BioLegend	108422	F (mm)/1:150

Rat Brilliant Violet 421 anti-mouse F4/80 mAb	BioLegend	123131	F (mm)/1:400
Rat Brilliant Violet 510 anti-Ly6C mAb	BioLegend	128033	F (mm)/1:40
Rat APC/Fire 750 anti-Ly6G mAb	BioLegend	127651	F (mm)/1:80
Rat BUV737 anti-CD8α mAb	BD Biosciences	564297	F (mm)/1:200
Rat FITC anti-CD4 mAb	BioLegend	100405	F (mm)/1:200
Rat Brilliant Violet 650 anti-CCR2 mAb	BioLegend	150613	F (mm)/1:60
Rat Alexa Fluor 647 anti-CD14 mAb	BioLegend	123328	F (mm)/1:200
Rat TruStain FcX PLUS (anti-CD16/32) mAb	BioLegend	156604	F (mm)/1:200
Gt anti-rat IgG (H+L) secondary Ab, Alexa Fluor 594 conjugated	Thermo Fisher Scientific	A-11007	IF (mm)/1:500
Gt anti-rb IgG (H+L) secondary Ab, Alexa Fluor 488 conjugated	Thermo Fisher Scientific	A-11034	IF (mm)/1:500
Gt anti-rb IgG (H+L) secondary Ab, Alexa Fluor 594 conjugated	Thermo Fisher Scientific	A-11012	IF (mm)/1:500

Abbreviations: mm=mouse; hs=human; gt=goat; rb=rabbit; mAb=monoclonal antibody; pAb=polyclonal antibody; IHC=immunohistochemistry; IF=immunofluorescence; F=flow cytometry; ISH=*in situ* hybridization; ChIP=chromatin immunoprecipitation

Supplementary Table 2. TaqMan expression probes' information for qPCR is summarized

Name	Company	Assay ID or Catalog number
Mouse Angptl4 (FAM/MGB)	Thermo Fisher Scientific	Mm00480431_m1
Mouse Ccl2 (FAM/MGB)	Thermo Fisher Scientific	Mm00441242_m1
Mouse Krt19 (CK19) (FAM/MGB)	Thermo Fisher Scientific	Mm00492980_m1
Mouse Amy1 (amylase) (FAM/MGB)	Thermo Fisher Scientific	Mm00651524_m1
Mouse Il6 (FAM/MGB)	Thermo Fisher Scientific	Mm00446190_m1
Mouse Tnf (FAM/MGB)	Thermo Fisher Scientific	Mm00443258_m1
Mouse Ywhaz (FAM/MGB)	Thermo Fisher Scientific	Mm01158417_g1
Mouse Ppard (FAM/ZEN/IBFQ)	Integrated DNA Technologies	Mm.PT.58.6994542
Mouse Actb (FAM/ZEN/IBFQ)	Integrated DNA Technologies	Mm.PT.51.9990212.g
Human ANGPTL4 (FAM/MGB)	Thermo Fisher Scientific	Hs01101125_m1
Human CCL2 (FAM/MGB)	Thermo Fisher Scientific	Hs00234140_m1
Human PPARD (FAM/ZEN/IBFQ)	Integrated DNA Technologies	Hs.PT.58.38841884
Human HPRT1 endogenous control (VIC/MGB)	Thermo Fisher Scientific	4326321E
