# Supplementary material for: Rapid acceleration of KRAS-mutant pancreatic carcinogenesis via remodeling of tumor immune microenvironment by PPARδ
Source: Nat Commun. 2022 May 13;13:2665. doi: 10.1038/s41467-022-30392-7 (PMC9106716; doi:10.1038/s41467-022-30392-7)
Supplement: Supplementary file 2 — Reporting Summary [file 41467_2022_30392_MOESM2_ESM.pdf]

nature portfolio

Corresponding author(s): Imad Shureiqi and Xiangsheng Zuo

Last updated by author(s): 04/15/2022

# Reporting Summary

Nature Portfolio wishes to improve the reproducibility of the work that we publish. This form provides structure for consistency and transparency in reporting. For further information on Nature Portfolio policies, see our Editorial Policies and the Editorial Policy Checklist.

## Statistics

For all statistical analyses, confirm that the following items are present in the figure legend, table legend, main text, or Methods section.

| n/a | Confirmed | |
|---|---|---|
| ☐ | ☒ | The exact sample size (*n*) for each experimental group/condition, given as a discrete number and unit of measurement |
| ☐ | ☒ | A statement on whether measurements were taken from distinct samples or whether the same sample was measured repeatedly |
| ☐ | ☒ | The statistical test(s) used AND whether they are one- or two-sided<br>*Only common tests should be described solely by name; describe more complex techniques in the Methods section.* |
| ☐ | ☒ | A description of all covariates tested |
| ☐ | ☒ | A description of any assumptions or corrections, such as tests of normality and adjustment for multiple comparisons |
| ☐ | ☒ | A full description of the statistical parameters including central tendency (e.g. means) or other basic estimates (e.g. regression coefficient) AND variation (e.g. standard deviation) or associated estimates of uncertainty (e.g. confidence intervals) |
| ☐ | ☒ | For null hypothesis testing, the test statistic (e.g. *F*, *t*, *r*) with confidence intervals, effect sizes, degrees of freedom and *P* value noted<br>*Give P values as exact values whenever suitable.* |
| ☒ | ☐ | For Bayesian analysis, information on the choice of priors and Markov chain Monte Carlo settings |
| ☒ | ☐ | For hierarchical and complex designs, identification of the appropriate level for tests and full reporting of outcomes |
| ☒ | ☐ | Estimates of effect sizes (e.g. Cohen's *d*, Pearson's *r*), indicating how they were calculated |

*Our web collection on statistics for biologists contains articles on many of the points above.*

## Software and code

Policy information about availability of computer code

| | |
|---|---|
| Data collection | 1. Digital HE staining slides for mouse pancreata tissues were scanned with Aperio AT2 (Leica biosystems).<br>2. Flow cytometry data for chemokine and cytokine panels were collected on BD FACS Canto II analyzer with FACSDiva software v 8.0 (BD). Flow cytometry data for immune cell profile were collected on LSR Fortessa X-20 analyzer with FACSDiva software v8.0 (BD). The cells were sorted on BD FACS Aria sorter with FACSDiva software v8.0 (BD).<br>3. qRT-PCR data were collected on StepOnePlus real time PCR systems (Applied biosystems) and analyzed with StepOne software.<br>4. mRNA-seq data was run and collected on an Illumina Hiseq 3000. |
| Data analysis | 1. The scanned HE slide images were captured with Aperio ImageScope software [v12.3.3.5048].<br>2. Flow cytometry data were analyzed by FlowJo v10 (BD).<br>3. Chemokine and cytokine panels were analyzed using LEGENDplex v8.0 Data Analysis Software (BioLegend).<br>4. RNA-seq analyses: After sequencing, the sequencing-generated BCL files were converted into fastq.gz files, and the sample libraries were de-multiplexed using CASAVA 1.8.2 to exclude mismatches. Genes were considered as differentially expressed genes using cut-off of p(Adi) <0.05. Gene set enrichment analyses were performed on differentially expressed genes using clusterProfiler package on R4.1.0 software to identify the signaling pathways.<br>5. All the statistical analyses were performed on SAS softwarev9.4 (SAS Institute) or GraphPad Prism v7.01 (GraphPad Software). |

For manuscripts utilizing custom algorithms or software that are central to the research but not yet described in published literature, software must be made available to editors and reviewers. We strongly encourage code deposition in a community repository (e.g. GitHub). See the Nature Portfolio guidelines for submitting code & software for further information.

## Data

Policy information about availability of data

All manuscripts must include a data availability statement. This statement should provide the following information, where applicable:

- Accession codes, unique identifiers, or web links for publicly available datasets
- A description of any restrictions on data availability
- For clinical datasets or third party data, please ensure that the statement adheres to our policy

The full RNA-seq data were deposited to the NCBI Gene Expression Omnibus public database with the accession # GSE176135 (https://www.ncbi.nlm.nih.gov/geo/query/acc.cgi?acc=GSE176135). Ppardelta mRNA-expression values of iKPC cell lines and iKPC orthotopic tumors (Supplementary Fig. 1j and k) were retrieved from the transcriptome-sequencing data in the NCBI Gene Expression Omnibus public database with #GSE32277 (https://www.ncbi.nlm.nih.gov/geo/query/acc.cgi?acc=GSE32277). Heatmap of human pancreatic chemokines' and cytokines' mRNA expression in pancreatic normal and PDAC tissues were retrieved from Badea pancreatic cancer data (https://www.ncbi.nlm.nih.gov/geo/query/acc.cgi?acc=gse15471) via Oncomine online tool (https://www.oncomine.org). The data for correlation between PPARD and CCL2 mRNA expression levels in human PDAC tissues were retrieved from Pan-Cancer Atlas public database analyses using TCGA data . All the other data are available within the article and its Supplementary Information. The remaining data are available within the article and its Supplementary Information. Source data are provided with this paper.

# Field-specific reporting

Please select the one below that is the best fit for your research. If you are not sure, read the appropriate sections before making your selection.

☒ Life sciences ☐ Behavioural & social sciences ☐ Ecological, evolutionary & environmental sciences

For a reference copy of the document with all sections, see nature.com/documents/nr-reporting-summary-flat.pdf

# Life sciences study design

All studies must disclose on these points even when the disclosure is negative.

| | |
|---|---|
| Sample size | The sample sizes for in vivo and in vitro studies were determined on the basis of our preliminary/pilot study results and further calculated with the guidance of our informatics coauthors Drs. Jing Wang and Xiaofeng Zheng. The sample sizes were chosen based on the adequacies to produce statistically differences among the experimental groups using similar experimental conditions on biological replicates in this study. |
| Data exclusions | No data were excluded for the experimental data analyses except for the experimental failures due to technical issues (e.g., failure of sample preparation, inappropriate usage of reagents and equipment). |
| Replication | All in-vitro studies and short term-treatment (i.e., 3 and 9 days GW) in-vivo animal studies with different genotypes were repeated 3 or more independent biological replicates under similar experimental conditions with similar results. For in vivo long-term treatment (12 or more weeks treatment) mouse experiments, we have worked with our informatics coauthors Drs. Jing Wang and Xiaofeng Zheng to ensure enough number sizes of animals with matched genetic background, age and sex that could produce statistically differences among the experimental groups in this study. We have used gain and loss of function experiments to double confirm the results in the long-term treatment animal experiments. |
| Randomization | Randomization of the mice were performed on the basis of the mouse body weights for the age- and sex- matched littermates with different genotypes. For the cell experiments, cells were randomly assigned to the different treatment groups. |
| Blinding | Human studies included no clinical trial or interventions to allow blinding. We did not consider blinding of animal studies because availability of information regarding the mouse genetic background and interventions to the research team and supporting animal care facility staff was necessary to accurately conduct studies. This information was also disclosed as required by the Institutional Animal Care and Use Committee rules to ensure the safety of the researchers and supporting veterinary team. The investigators were also not blinded for in vitro cell experiments to practically conduct the experiments. |

# Reporting for specific materials, systems and methods

We require information from authors about some types of materials, experimental systems and methods used in many studies. Here, indicate whether each material, system or method listed is relevant to your study. If you are not sure if a list item applies to your research, read the appropriate section before selecting a response.

## Materials & experimental systems

| n/a | Involved in the study | |
|---|---|---|
| ☐ | ☒ | Antibodies |
| ☐ | ☒ | Eukaryotic cell lines |
| ☒ | ☐ | Palaeontology and archaeology |
| ☐ | ☒ | Animals and other organisms |
| ☐ | ☒ | Human research participants |
| ☒ | ☐ | Clinical data |
| ☒ | ☐ | Dual use research of concern |

## Methods

| n/a | Involved in the study | |
|---|---|---|
| ☒ | ☐ | ChIP-seq |
| ☐ | ☒ | Flow cytometry |
| ☒ | ☐ | MRI-based neuroimaging |

# Antibodies

Antibodies used

All the antibodies used in this study were summarized in a table for their sources, catalog numbers, applications and dilutions in Methods section "Antibodies".
Rb anti–α-amylase pAb, Sigma-Aldrich, A8273, IF (mm): 1:250
Rat anti-CK19 mAb, Developmental Studies Hybridoma Bank, TROMA-III-c,IF (mm): 1:100
Mm anti–α-SMA mAb, Sigma-Aldrich, A5228, IF (mm): 1:50
Mm anti-DDK mAb, OriGene, TA50011-100, ChIP (mm): 1:200/WB (hs/mm): 1:2000
Mm anti-PPARD mAb, Santa Cruz Biotechnology, sc-74517X, ChIP (hs/mm):1:500
Normal mouse IgG, Santa Cruz Biotechnology, sc-2025, ChIP (hs/mm):1:100
Rb anti–phospho-ERK1/2 mAb, Cell Signaling Technology, 4370s, IHC (mm): 1:400, WB (hs/mm):1:2000
Rb anti-phospho-Stat3 (Tyr705) mAb, Cell Signaling Technology, 9145s, IHC (mm):1:200, WB (hs/mm): 1:1000
Rb anti-Ppard pAb, Abcam, Ab8937, WB (mm): 1:750
Mm anti-Ras mAb in active Ras detection kit (8821), Cell Signaling Technology, WB (mm): 1:200
Mm anti–β-Actin mAb, Santa Cruz Biotechnology, sc-47778, WB (hs/mm): 1:1000
Rb anti-PPARD pAb, Aviva Systems Biology, ARP38765_T100, IHC (hs/mm): 1:100
Rat anti-F4/80 mAb, Cell Signaling Technology, 71299S IF (mm): 1:100
Rb anti-F4/80 mAb, Cell Signaling Technology, 70076S IHC (mm): 1:200, Dual ISH-IHC (mm): 1:200
Rb anti-CCR2 mAb, Abcam, Ab273050 IF (mm): 1:250, Dual ISH-IF (mm): 1:250
Rat anti-Gr1 mAb, BioLegend, 108402, IF (mm): 1:50
Rat PE anti-IA/IE mAb, BioLegend 107607, F (mm): 1:500
Rat PerCP/Cy5.5 anti-CD45 mAb, BioLegend, 103131, F (mm) :1:150
Rat Brilliant Violet 785 anti-CD3ε mAb, BioLegend, 100355, F (mm):1:60
Rat Alexa Fluor 700 anti- CD45R/B220 mAb, BioLegend,103231, F (mm):1:200
Rat PE/Cy7 anti-CD11b mAb, BioLegend, 101215 F (mm):1:250
Rat Alexa Fluor 700 anti-Ly-6G/Ly-6C (Gr-1) mAb BioLegend 108422 F (mm):1:150
Rat Brilliant Violet 421 anti-mouse F4/80 mAb, BioLegend, 123131, F (mm):1:400
Rat Brilliant Violet 510 anti-Ly6C mAb, BioLegend, 128033, F (mm):1:40
Rat APC/Fire 750 anti-Ly6G mAb, BioLegend, 127651, F (mm):1:80
Rat BUV737 anti-CD8α mAb, BD Biosciences, 564297, F (mm):1:200
Rat FITC anti-CD4 mAb, BioLegend 100405, F (mm):1:200
Rat Brilliant Violet 650 anti-CCR2 mAb, BioLegend, 150613, F (mm):1:60
Rat Alexa Fluor 647 anti-CD14 mAb, BioLegend 123328, F (mm):1:200
Rat TruStain FcX PLUS (anti-CD16/32) mAb, BioLegend, 156604, F (mm):1:200
Gt anti-rat IgG (H+L) secondary Ab, Alexa Fluor 594 conjugated, Thermo Fisher Scientific, A-11007 IF (mm):1:500
Gt anti-rb IgG (H+L) secondary Ab, Alexa Fluor 488 conjugated, Thermo Fisher Scientific, A-11034 IF (mm) :1:500
Gt anti-rb IgG (H+L) secondary Ab, Alexa Fluor 594 conjugated, Thermo Fisher Scientific, A-11012 IF (mm) :1:500

Abbreviations: mm=mouse; hs=human; gt=goat; rb=rabbit; mAb=monoclonal antibody; pAb=polyclonal antibody; IHC=immunohistochemistry; IF=immunofluorescence; F=flow cytometry; ISH=in situ hybridization; ChIP=chromatin immunoprecipitation

Validation

All antibodies were validated by the suppliers for the applications in their data sheets or manufacturers' websites that we used in this study, and were checked in our lab by comparing to vendors' results. For the antibodies which had multiple bands for Western blot, we have added positive control along with the experimental samples.
Rb anti–α-amylase pAb (A8273): https://www.sigmaaldrich.com/US/en/product/sigma/a8273
Rat anti-CK19 mAb (TROMA-III-c):https://dshb.biology.uiowa.edu/TROMA-III
Mm anti–α-SMA mAb (A5228): https://www.sigmaaldrich.com/US/en/product/sigma/a5228
Mm anti-DDK mAb (TA50011-100):https://www.origene.com/catalog/antibodies/tag-antibodies/ta50011-100/clone-oti4c5-anti-ddk-flag-monoclonal-antibody
Mm anti-PPARD mAb (sc-74517X): https://datasheets.scbt.com/sc-74517.pdf
Normal mouse IgG (sc-2025): https://datasheets.scbt.com/sc-2025.pdf
Rb anti–phospho-ERK1/2 mAb (4370s): https://www.cellsignal.com/products/primary-antibodies/phospho-p44-42-mapk-erk1-2-

thr202-tyr204-d13-14-4e-xp-rabbit-mab/4370
Rb anti-phospho-Stat3 (Tyr705) mAb (9145s): https://www.cellsignal.com/products/primary-antibodies/phospho-stat3-tyr705-d3a7-xp-rabbit-mab/9145
Rb anti-Ppard pAb (Ab8937): https://www.abcam.com/ppar-delta-antibody-ab8937.html
Mm anti-Ras mAb in active Ras detection kit (8821): https://www.cellsignal.com/products/cellular-assay-kits/active-ras-detection-kit/8821
Mm anti–β-Actin mAb (sc-47778): https://datasheets.scbt.com/sc-47778.pdf
Rb anti-PPARD pAb (ARP38765_T100): https://www.avivasysbio.com/ppard-antibody-c-terminal-region-arp38765-t100.html
Rat anti-F4/80 mAb (71299s): https://www.cellsignal.com/products/primary-antibodies/f4-80-bm8-1-rat-mab/71299
Rb anti-F4/80 mAb (70076s): https://www.cellsignal.com/products/primary-antibodies/f4-80-d2s9r-xp-rabbit-mab/70076
Rb anti-CCR2 mAb (Ab273050): https://www.abcam.com/ccr2-antibody-epr20844-15-ab273050.html
Rat anti-Gr1 mAb (108402): https://www.biolegend.com/en-us/products/purified-anti-mouse-ly-6g-ly-6c-gr-1-antibody-462
Rat PE anti-IA/IE mAb (107607): https://www.biolegend.com/en-us/products/pe-anti-mouse-i-a-i-e-antibody-367
Rat PerCP/Cy5.5 anti-CD45 mAb (103131): https://www.biolegend.com/en-us/products/percp-cyanine5-5-anti-mouse-cd45-antibody-4264
Rat Brilliant Violet 785 anti-CD3ε mAb (100355): https://www.biolegend.com/en-us/products/brilliant-violet-785-anti-mouse-cd3epsilon-antibody-12081
Rat Alexa Fluor 700 anti-CD45R/B220 mAb (103231):https://www.biolegend.com/en-us/products/alexa-fluor-700-anti-mouse-human-cd45r-b220-antibody-3408
Rat PE/Cy7 anti-CD11b mAb (101215): https://www.biolegend.com/en-us/products/pe-cyanine7-anti-mouse-human-cd11b-antibody-1921
Rat Alexa Fluor 700 anti-Ly-6G/Ly-6C (Gr-1) mAb (108422): https://www.biolegend.com/en-us/products/alexa-fluor-700-anti-mouse-ly-6g-ly-6c-gr-1-antibody-3390
Rat Brilliant Violet 421 anti-mouse F4/80 mAb (123131): https://www.biolegend.com/en-us/products/brilliant-violet-421-anti-mouse-f4-80-antibody-7199
Rat Brilliant Violet 510 anti-Ly6C mAb (128033): https://www.biolegend.com/en-us/products/brilliant-violet-510-anti-mouse-ly-6c-antibody-8726
Rat APC/Fire 750 anti-Ly6G mAb (127651): https://www.biolegend.com/en-us/products/apc-fire-750-anti-mouse-ly-6g-antibody-13198
Rat BUV737 anti-CD8α mAb BD (564297): https://www.bdbiosciences.com/en-nz/products/reagents/flow-cytometry-reagents/research-reagents/single-color-antibodies-ruo/buv737-rat-anti-mouse-cd8a.612759
Rat FITC anti-CD4 mAb (100405): https://www.biolegend.com/en-us/products/fitc-anti-mouse-cd4-antibody-248
Rat Brilliant Violet 650 anti-CCR2 mAb (150613): https://www.biolegend.com/en-us/products/brilliant-violet-650-anti-mouse-cd192-ccr2-antibody-15387
Rat Alexa Fluor 647 anti-CD14 mAb (123328): https://www.biolegend.com/en-us/products/alexa-fluor-647-anti-mouse-cd14-antibody-15102
Rat TruStain FcX PLUS (anti-CD16/32) mAb (156604): https://www.biolegend.com/en-us/products/trustain-fcx-plus-anti-mouse-cd16-32-antibody-17085
Gt anti-rat IgG (H+L) secondary Ab, Alexa Fluor 594 conjugated (A-11007): https://www.thermofisher.com/antibody/product/Goat-anti-Rat-IgG-H-L-Cross-Adsorbed-Secondary-Antibody-Polyclonal/A-11007
Gt anti-rb IgG (H+L) secondary Ab, Alexa Fluor 488 conjugated (A-11034): https://www.thermofisher.com/antibody/product/Goat-anti-Rabbit-IgG-H-L-Highly-Cross-Adsorbed-Secondary-Antibody-Polyclonal/A-11034
Gt anti-rb IgG (H+L) secondary Ab, Alexa Fluor 594 conjugated (A-11012): https://www.thermofisher.com/antibody/product/Goat-anti-Rabbit-IgG-H-L-Cross-Adsorbed-Secondary-Antibody-Polyclonal/A-11012

## Eukaryotic cell lines

Policy information about cell lines

| | |
|---|---|
| Cell line source(s) | One NB490 KPC, two iKPC, and two KPC mouse PDAC cell lines were kindly provided by Drs. Anirban Maitra and Haoqiang Ying at MD Anderson Cancer Center and by David A. Tuveson at Cold Spring Harbor Laboratory, respectively. KC and KC/Pd mouse PDAC cell lines were generated by our laboratory. L3.3, L3.9, Colo357, Panc-1, PAU8902, and MDA48 human PDAC cells were kindly provided by Dr. Isaiah Fidler's laboratory. |
| Authentication | Mouse and human PDAC cells generated by our lab or from other labs as gifts were authenticated by measurements of pancreatic acinar and ductal markers (amylase and CK19), morphology, proliferation in vitro and tumorogenicity in vivo. Authenticity for the cells from ATCC was based on ATCC disclosure. |
| Mycoplasma contamination | All cell lines were tested negative for mycoplasma contamination by using PCR method. |
| Commonly misidentified lines (See ICLAC register) | No misidentified cell lines were used in this study. |

## Animals and other organisms

Policy information about studies involving animals; ARRIVE guidelines recommended for reporting animal research

| | |
|---|---|
| Laboratory animals | All the mice used in this study were clearly described in the Methods sections "Animals and Generation of experimental mice": C57BL/6J genetic background |

| | |
|---|---|
| | Transgenic Panc-pd, KC, KC/pd, KC/pdKO, KC/tdPd mice<br>Age from 6 weeks to 34 weeks<br>Gender: female and male both were included.<br>The mice were housed with a dark/light cycle of 12 h, ambient temperature of 22C and humidity of 30-70%. |
| Wild animals | Wild animals were not used in the study. Only inbred mice were used as described in the Methods sections "Animals and Generation of experimental mice". |
| Field-collected samples | No field collected samples were used in the study. |
| Ethics oversight | The ACUF protocols for all animal experiments were approved by and performed at MDACC in accordance to the MDACC Animal Ethical guidelines. |

Note that full information on the approval of the study protocol must also be provided in the manuscript.

## Human research participants

Policy information about studies involving human research participants

| | |
|---|---|
| Population characteristics | A set of pancreatic tissue microarrays including paired normal tissue, PanINs, and PDAC were purchased from US Biomax (#BIC14011a).<br>Human pancreatic tissue samples were collected from two patients who underwent endoscopic ultrasound guided biopsies of pancreatic neoplastic lesions at MD Anderson Cancer Center and from nine patients who underwent surgical resection of pancreatic neoplastic lesions at Rogel Cancer Center, University of Michigan. The current study using these tissue samples was approved by The Institutional Review Boards of MD Anderson and University of Michigan. De-identified sections from freshly formalin-fixed pancreatic tissues were used for this study. No clinical co-variate information was available because of the de-identified nature of the samples. |
| Recruitment | Materials used for this study were residual pancreatic tissues from biopsies or surgical resection of pancreatic neoplastic lesions for patients' routine care. No new recruitment was required in this study. |
| Ethics oversight | MDACC and University of Michigan Institutional human ethics committee approved the conduct of reported studies of human tissue samples. Informed consents were obtained from participants at both MDACC and University of Michigan. |

Note that full information on the approval of the study protocol must also be provided in the manuscript.

## Flow Cytometry

### Plots

Confirm that:

- [x] The axis labels state the marker and fluorochrome used (e.g. CD4-FITC).
- [x] The axis scales are clearly visible. Include numbers along axes only for bottom left plot of group (a 'group' is an analysis of identical markers).
- [x] All plots are contour plots with outliers or pseudocolor plots.
- [x] A numerical value for number of cells or percentage (with statistics) is provided.

### Methodology

| | |
|---|---|
| Sample preparation | Immune cell profiling<br>Mouse pancreatic tissues were digested by digestion buffer at 37°C for 30 min. The digested tissues were pushed through a 70-µm cell strainer and washed by 40 ml of DMEM without FBS. The digested cells were re-suspended in 37% Percoll, with the same volume of 70% Percoll on the bottom of the tube. After centrifugation at 800 ×g for 20 min with break off, the immune cells were isolated from the 37%/70% interface and rinsed by PBS. The cells were first stained by Zombie UV (BioLegend, #423107), then were incubated with a cocktail comprising the following antibodies in cell staining buffer (#420201, BioLegend): anti-CD45 (#103131), anti-CD3ε (#100355), anti-B220 (#103231), anti-Gr1 (#108438), anti-CD11b (#101215), anti-F4/80 (#123131), anti-Ly6C (#128033), anti-Ly6G (#127651), anti-CD4 (#100405), anti-IA/IE (#107607), anti-CD14 (#123328), all from BioLegend, and anti-CD8a (#564297, BD Biosciences). After washing, the stained cells were submitted for multiple-color flow cytometry analysis.<br><br>tdTomato RFP cell sorting<br>Mouse pancreatic tissues from KC/tdPd mice fed a GW501516 (50 mg/kg) or control diet for 3 days were rinsed by cold PBS, cut into 1-mm pieces, and incubated with digestion buffer at 37°C for 30 min. The digested tissues were passed through a 70-µm cell strainer, washed by DMEM, and re-suspended in DMEM containing 1% fetal bovine serum and 2 mM EDTA. Digested cells were then sorted by flow cytometry using td-Tomato RFP and harvested in the fresh complete DMEM medium.<br><br>MDSCs sorting<br>To isolate immune cells before sorting, we first harvested spleens from adult C57/BL6 mice and cut the spleens into small pieces (~1 mm). Then we used the plunger base of a syringe to mash the spleens to pass through a 70-µm cell strainer while washing with RPMI medium (10% FBS) until the spleen was completely mashed. The cells were spun down at 350 ×g for 3 min, and the cell pellets were resuspended in 5mL red blood cell lysis buffer (BioLegend) on ice for 5 min, then 40 ml of cold |

PBS was added and spun down at 350 ×g for 3 min. The cells were washed with cold PBS once and spun down before being stained with rat PE/Cy7 monoclonal anti-mouse/human CD11b and rat Alexa Fluor 700 anti-mouse Ly-6G/Ly-6C (Gr-1) (BioLegend). CD11b+/Gr1+ cells (MDSCs) were sorted by flow cytometry and harvested for the chemoattractant assay.

Beads based chemokine and cytokine panels flow cytometry
Two LEGENDplex beads based assay kits including chemokines (BioLegend, #740007) or cytokines (BioLegend, #740150) were used. The protein lysate was prepared as followed: soon after mice were euthanized, their pancreatic tissues were flash-frozen by liquid nitrogen in sterile tubes. The frozen tissues were grounded into powder in liquid nitrogen-chilled aluminum foil and mechanically homogenized in SDS free protein lysis buffer. The protein samples were diluted and performed beads binding and PE staining according to the manufacturer's manual.

| | |
|---|---|
| Instrument | FACS Canto II analyzer (BD), LSR Fortessa X-20 analyzer (BD), FACS Aria (BD) |
| Software | FACSDiva software v8.0 (BD), FlowJo software v10 (BD), LEGENDplex v8.0 Data Analysis Software |
| Cell population abundance | Sorted tdTomato positive pancreatic cell population with purity >90% was determined by manually counting under fluorescence microscope. CD11b+/Gr1+ MDSC population with purity >98% was determined by the post-sort fraction. |
| Gating strategy | Gating strategy for immune cell profile: Stained tissue infiltrating immune cells were gated based on SSC and FCS, and further gated for live cells. Then live cells were gated on CD45+ cells and further on a series of surface markers for different immune cells.<br><br>FACS sorting strategy of tdTomato+ pancreatic epithelial cells and spleen MDSCs: For tdTomato+ cells, digested cells were firstly gated by SSC and FCS, and then gated on tdTomato+ cells. Purity of cells were determined by the observation under fluorescence microscope. For mouse spleenic MDSCs, CD11b and Gr1 stained spleen cells were gated on size discrimination, doublet exclusion (FSC-H vs FSC-W and SSC-H vs SSC-W), and then gated on live cells. Live cells were further sorted based on CD11b+/Gr1+ gate. Purity of cells were determined by post-sort fraction.<br><br>Chemokine and cytokine panel gating strategy: The chemokines or cytokines binding beads were gated based on SSC and FCS (linear mode) to determine beads A and beads B. Then both of beads A and beads B were gated on APC and PE in log mode. The result gatings and calculations were performed on LEGENDplex Data Analysis Software according to the manufacturer's manual. |

☒ Tick this box to confirm that a figure exemplifying the gating strategy is provided in the Supplementary Information.
